# An Immunomodulatory Zinc‐Alum/Ovalbumin Nanovaccine Boosts Cancer Metalloimmunotherapy Through Erythrocyte‐Assisted Cascade Immune Activation

**DOI:** 10.1002/advs.202307389

**Published:** 2023-12-08

**Authors:** Jing Zhao, Lingxiao Zhang, Pin Li, Shanbiao Liu, Shiyi Yu, Zheng Chen, Mingjian Zhu, Shangzhi Xie, Daishun Ling, Fangyuan Li

**Affiliations:** ^1^ Institute of Pharmaceutics Hangzhou Institute of Innovative Medicine College of Pharmaceutical Sciences Zhejiang University Hangzhou 310058 P. R. China; ^2^ Interdisciplinary Nanoscience Center (iNANO) Aarhus University Aarhus C DK‐8000 Denmark; ^3^ Frontiers Science Center for Transformative Molecules School of Chemistry and Chemical Engineering School of Biomedical Engineering National Center for Translational Medicine Shanghai Jiao Tong University Shanghai 200240 P. R. China; ^4^ World Laureates Association (WLA) Laboratories Shanghai 201203 P. R. China; ^5^ Key Laboratory of Precision Diagnosis and Treatment for Hepatobiliary and Pancreatic Tumor of Zhejiang Province Hangzhou 310009 P. R. China

**Keywords:** cancer metalloimmunotherapy, erythrocytes, nanovaccines, spleen, tumor microenvironment

## Abstract

Cancer therapeutic vaccines are powerful tools for immune system activation and eliciting protective responses against tumors. However, their efficacy has often been hindered by weak and slow immune responses. Here, the authors introduce an immunization strategy employing senescent erythrocytes to facilitate the accumulation of immunomodulatory zinc‐Alum/ovalbumin (ZAlum/OVA) nanovaccines within both the spleen and solid tumors by temporarily saturating liver macrophages. This approach sets the stage for boosted cancer metalloimmunotherapy through a cascade immune activation. The accumulation of ZAlum/OVA nanovaccines in the spleen substantially enhances autophagy‐dependent antigen presentation in dendritic cells, rapidly initiating OVA‐specific T‐cell responses against solid tumors. Concurrently, ZAlum/OVA nanovaccines accumulated in the tumor microenvironment trigger immunogenic cell death, leading to the induction of individualized tumor‐associated antigen‐specific T cell responses and increased T cell infiltration. This erythrocyte‐assisted cascade immune activation using ZAlum/OVA nanovaccines results in rapid and robust antitumor immunity induction, holding great potential for clinical cancer metalloimmunotherapy.

## Graphical abstract

An immunomodulatory zinc‐Alum/ovalbumin nanovaccine, combined with an innovative immunization strategy that utilizes senescent erythrocytes to enhance accumulation in both the spleen and tumors, has been introduced. This collaborative spatial immunization, which connects the spleen and the tumor microenvironment, enhances antitumor immunity through a cascade immune activation and increased T cell infiltration, offering promising prospects for clinical cancer metalloimmunotherapy.

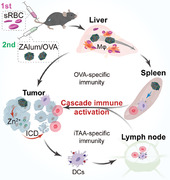

## 1 Introduction

Cancer therapeutic vaccines have emerged as a promising frontier in cancer treatment, harnessing tumor‐specific immunity and enabling durable immune surveillance.^[^ ^1^ ^]^ Despite the potential benefits, their therapeutic efficacy in clinical settings remains challenging.^[^ ^2^ ^]^ Primary obstacles lie in the slow immune induction and the difficulty of triggering robust immune responses.^[^ ^3^ ^]^ Spatial control of vaccine delivery is crucial for effectively inhibiting tumor proliferation through antitumor immune responses.^[^ ^4^ ^]^ The spleen, being the largest secondary lymphoid organ housing a dense population of antigen‐presenting cells (APCs) and B/T cells,^[^ ^5^ ^]^ presents a target site for intravenously injected vaccines, ensuring rapid and potent immune responses.^[^ ^6^ ^]^ However, the clearance of most circulating vaccines by liver macrophages significantly curtails their activity.^[^ ^7^ ^]^ Previous studies have drawn inspiration from the intrinsic spleen‐targeting ability of erythrocytes (also known as red blood cells, RBCs) and explored various biomimetic strategies, such as encapsulating antigens within RBCs, employing surface loading (cell hitchhiking), or fusing antigens into RBC membranes, to achieve spleen‐targeted vaccine delivery and enhance T cell activation.^[^ ^8^ ^]^ Nevertheless, despite these improvements, the T cell infiltration into the immunologically cold tumor microenvironment (TME) remains restricted.^[^ ^9^ ^]^ Interestingly, host‐derived senescent RBCs (sRBCs) are physiologically captured by both liver and spleen macrophages,^[^ ^10^ ^]^ leading to transient saturation of macrophages and a subsequent reduction in their ability to uptake nanoparticles.^[^ ^11^ ^]^ Thus, pre‐administration of sRBCs effectively prolongs nanoparticle circulation and enhances their accumulation in the spleen.^[^ ^10^, ^11^ ^]^ Additionally, neutralizing liver macrophages beforehand enhances nanoparticle accumulation in tumors, laying the foundation for direct TME regulation.^[^ ^12^ ^]^

On the other hand, vaccine adjuvants play a crucial role in bolstering antigen‐specific immune responses, but conventional approved adjuvants often fall short due to their poor adjuvanticity in cancer immunotherapy.^[^ ^13^ ^]^ Notably, nutritional metal ions can be used as novel immunomodulators for cancer immunotherapy (termed metalloimmunotherapy).^[^ ^14^ ^]^ Accumulating evidence supports the role of zinc ions (Zn^2+^) in activating the autophagy signaling pathway, facilitating antigen cross‐presentation by dendritic cells (DCs), and inducing T‐cell activation.^[^ ^15^ ^]^ Moreover, Zn^2+^ induces toxic oxidative stress in tumor cells, leading to cell apoptosis,^[^ ^16^ ^]^ and triggering immunogenic cell death (ICD), consequently increasing T‐cell infiltration.^[^ ^17^ ^]^ Furthermore, ICD can transform the primary tumor into an antigen depot, and the released individualized tumor‐associated antigens (iTAAs) facilitate the activation of iTAA‐specific T cells.^[^ ^1^, ^18^ ^]^ Given the dual role of Zn^2+^ in promoting immune activation and triggering ICD, incorporating Zn^2+^ into Alum (the only inorganic adjuvant approved by the US Food and Drug Administration) may potentiate cancer metalloimmunotherapy through enhanced T cell immune responses.

**Scheme 1:**
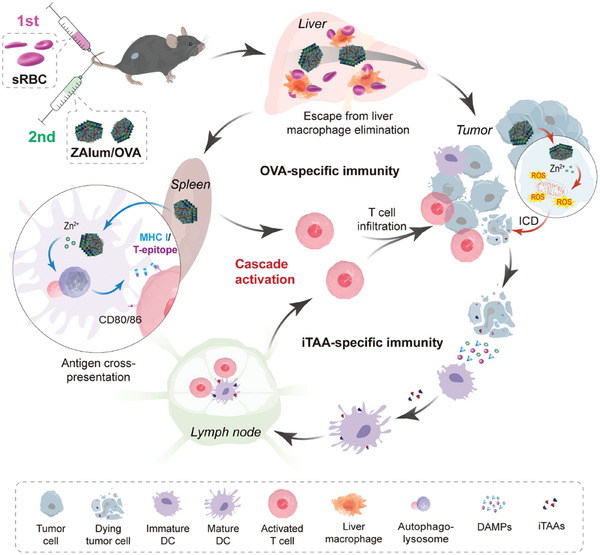
Erythrocyte‐assisted systemic immunization of ZAlum/OVA nanovaccines promoting potent cancer metalloimmunotherapy through cascade immune activation. This process entails the administration of ZAlum/OVA nanovaccines 16 h subsequent to sRBCs injection, strategically neutralizing liver macrophages. This deliberate intervention facilitates the accumulation of immunomodulatory ZAlum/OVA nanovaccines within both the spleen and the TME. Within the spleen, ZAlum/OVA nanovaccines exhibit efficient autophagy‐dependent antigen cross‐presentation, culminating in robust activation of OVA‐specific T cells. Concurrently, within the TME, ZAlum/OVA nanovaccines invoke ICD, thereby facilitating iTAA‐specific T cells and augmented T cell infiltration. By seamlessly amalgamating these concerted actions, the erythrocyte‐assisted systemic immunization of ZAlum/OVA nanovaccines introduces a promising trajectory toward efficient cancer metalloimmunotherapy.

In light of these considerations, herein, we report on the designed fabrication of immunomodulatory zinc‐Alum/ovalbumin (ZAlum/OVA) nanovaccines, and present an erythrocyte‐assisted systemic immunization strategy for ZAlum/OVA nanovaccines, effectively promoting rapid and potent antitumor immunity through cascade immune activation. By pre‐injecting sRBCs to transiently saturate liver macrophages and subsequently administering ZAlum/OVA nanovaccines systemically, we achieve simultaneous accumulation of the nanovaccines in both the spleen and the tumor. In the spleen, ZAlum/OVA nanovaccines induce efficient autophagy‐dependent antigen cross‐presentation, promoting OVA‐specific T‐cell activation. Concurrently, in the TME, ZAlum/OVA nanovaccines trigger ICD, activating iTAA‐specific T cells and enhancing T cell infiltration. This erythrocyte‐assisted cascade immune activation approach, as outlined in **Scheme** **1**, offers a straightforward yet effective strategy for robust cancer metalloimmunotherapy.

## 2 Results and Discussion

### 2.1 Synthesis and Characterization of ZAlum and ZAlum/OVA

**Figure 1:**
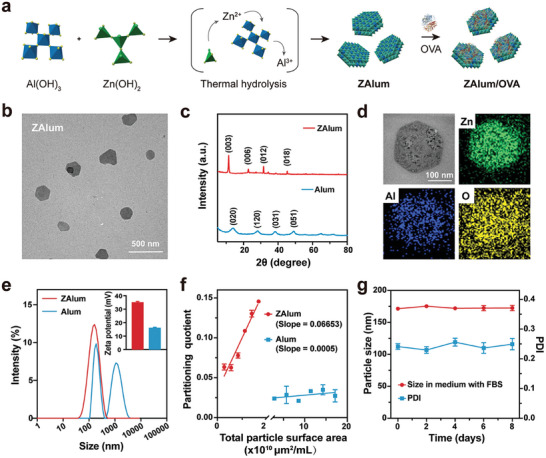
Synthesis and characterizations of ZAlum adjuvant. a) Schematic illustration of the construction of ZAlum and ZAlum/OVA. b) TEM image of ZAlum. Scale bar = 500 nm. c) XRD patterns (Cu Kα radiation, from 5° to 80°) of ZAlum (red) and Alum (blue). a.u., arbitrary units. d) EDS mapping of a single ZAlum. Scale bar = 100 nm. e) Particle size distribution and Zeta potential (inset) of ZAlum (red) and Alum (blue) were elucidated via dynamic light scattering. f) Evaluation of relative hydrophobicity through Rose Bengal adsorption onto ZAlum (red) and Alum (blue). g) Colloidal stability of ZAlum/OVA nanovaccines in medium with 10% FBS by monitoring the particle size over time. Data are presented as the mean ± s.d. (*n* = 3).

The meticulous synthesis and thorough characterization of ZAlum underpin their potential as impactful adjuvants. ZAlum adjuvant was obtained from a mixture of Al(OH)_3_ (a pivotal Alum component) and Zn(OH)_2_ through hydrothermal hydrolysis.^[^ ^19^ ^]^ The subsequent ultrasound treatment facilitated the loading of the model antigen OVA onto the surface of ZAlum,^[^ ^20^ ^]^ culminating in the formation of the ZAlum/OVA nanovaccines (**Figure** **1**a). Our method encompassed an optimization phase, where diverse initial Zn/Al precursor molar ratios were evaluated. A stable and uniform ZAlum adjuvant with a hexagonally‐shaped structure was acquired at an initial Zn/Al precursor molar ratio of 2:1 (Figure 1b; Figure S1, Supporting Information). Furthermore, the observed formation of a hydrotalcite‐like structure, corroborated by X‐ray diffraction (XRD) patterns, attests to the successful synthesis (JCPDS No. 38–0486) (Figure 1c),^[^ ^21^ ^]^ and the Zn/Al molar ratio (2.09 ± 0.28) closely approximated the initial ratio (Table S1, Supporting Information).

Alum (a commercial adjuvant purchased from Invivogen) comprises aggregates of rod‐shaped nanocrystals (Figure S2, Supporting Information).^[^ ^22^ ^]^ They elicit weak cellular immune responses against tumors due to abortive phagocytosis by APCs,^[^ ^23^ ^]^ and limited transport into the lymphatic system accompanied by the necessitated localized administration.^[^ ^3a^ ^]^ Compared to Alum, ZAlum adjuvant successfully introduces zinc into the layers, which is demonstrated by energy dispersive spectrum (EDS) elemental mapping (Figure 1d). Interestingly, the ZAlum adjuvant manifests an average particle size of 146.0 ± 1.5 nm with a polydispersity index (PDI) of 0.173, which renders it particularly amenable for systemic injection and delivery (Figure 1e). Moreover, the discernible hydrophobicity which contributed to cellular uptake^[^ ^24^ ^]^ was verified using Rose Bengal (a hydrophobic dye).^[^ ^25^ ^]^ The slope of the partitioning quotient against total surface area (calculated by surface area in Table S2, Supporting Information) indicates a higher hydrophobicity of ZAlum (slope = 0.06653) compared to Alum (slope = 0.0005) (Figure 1f). Such small uniform size and conspicuous hydrophobicity facilitate efficient vaccine delivery, including suitability for systemic injection and heightened phagocytosis by APCs.

The positively charged surface of ZAlum (+35.1 ± 0.8 mV) and Alum adjuvants (+16.0 ± 0.6 mV) can readily adsorb OVA onto their surfaces to obtain ZAlum/OVA and Alum/OVA nanovaccines (Figure 1e; Figure S3a, Supporting Information). Transmission electron microscope (TEM) images illustrate the uniform adsorption of OVA onto both ZAlum and Alum adjuvants (Figure S3b,c, Supporting Information). Fourier transform infrared spectra, along with hydrodynamic size measurements and Zeta potential analyses, collectively confirm the successful adsorption. The ZAlum/OVA nanovaccine exhibits a hydrodynamic size of 177 ± 5.5 nm with a PDI of 0.158 and undergoes a change in surface charge from positive to negative (−9.9 ± 0.1 mV) (Figure S3d‐f, Supporting Information). Furthermore, ZAlum/OVA nanovaccines maintain a similar size during storage over time in a medium containing 10% fetal bovine serum (FBS) (Figure 1g), indicating excellent colloidal stability. The hemolysis assay indicates that ZAlum/OVA nanovaccines have a negligible hemolytic effect, suggesting excellent blood compatibility (Figure S4, Supporting Information).

We then turned our attention to the release of Zn^2+^ from ZAlum/OVA nanovaccines (Table S3, Supporting Information). These nanovaccines were exposed to PBS at varying pH levels (5.5, 6.5, and 7.4), and the concentration of Zn^2+^ was assessed through inductively coupled plasma mass spectrometry (ICP‐MS) following a 24‐h incubation. The findings unveil a 5.35‐fold increment in the release of Zn^2+^ from ZAlum/OVA nanovaccines in pH 5.5 PBS as compared to pH 6.5 PBS, with near‐negligible release observed in pH 7.4 PBS, indicating the pH‐responsive nature of Zn^2+^ release from ZAlum/OVA nanovaccines. Besides, the release of Al^3+^ remains negligible across diverse buffer conditions. These results suggest that the ZAlum/OVA nanovaccine can effectively release Zn^2+^, which can act as an immunostimulatory for cancer metalloimmunotherapy.

### 2.2 Erythrocyte‐Assisted Systemic Delivery of ZAlum/OVA Nanovaccines for Simultaneous Spleen and Tumor Accumulation

**Figure 2:**
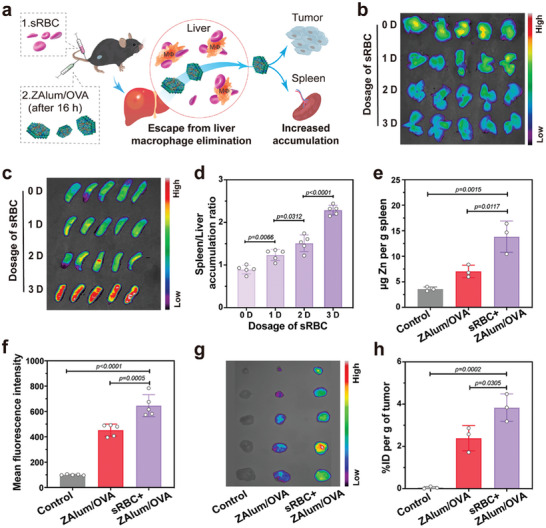
Erythrocyte‐assisted systemic delivery enables dual accumulation of ZAlum/OVA nanovaccines in the spleen and tumor. a) Schematic illustrating the erythrocyte‐assisted systemic delivery approach, resulting in the accumulation of ZAlum/OVA nanovaccines in both the spleen and tumor. b,c) Representative ex vivo fluorescent images of livers (b) and spleens (c) at 24 h preinjected with different dosages of sRBCs (0, 1, 2 and 3 D) before Cy5‐labelled ZAlum/OVA nanovaccines administration. 1 D = 2×10^8^ cells. d) Spleen/liver accumulation ratios were computed based on fluorescence intensity data from (b) and (c) (*n* = 5). e) The mass of Zn per g of spleen after ZAlum/OVA nanovaccine injection with or without 3 D sRBCs pre‐injection (*n* = 3). f) Fluorescence quantification of tumors in (g) (*n* = 5). (g) Representative ex vivo fluorescent images of tumors at 24 h after i.v. injection of Cy5‐labelled ZAlum/OVA nanovaccines with or without 3 D sRBCs pre‐injection. h) The % ID per g of tumors after i.v. injection of ZAlum/OVA nanovaccines with or without sRBCs pre‐injection (*n* = 3). Data are presented as the mean ± s.d. Statistical significance was tested by one‐way ANOVA with Tukey's post hoc test.

To achieve spatial control of nanovaccine delivery, a strategic injection of sRBCs was orchestrated to transiently neutralize liver macrophages, thus the subsequently injected ZAlum/OVA nanovaccines can avoid the liver clearance to accumulate simultaneously in the spleen and tumor (**Figure** **2**a). To examine the effect of erythrocyte‐assisted approach on the in vivo distribution of ZAlum/OVA nanovaccines, Cy5‐labelled ZAlum/OVA nanovaccines were intravenously administered to tumor‐bearing mice 16 h after the pre‐injection of sRBCs at varying dosages (0, 1, 2, and 3 D).^[^ ^10b^ ^]^ Fluorescence intensities of the liver and spleen were evaluated after 24 h (Figure 2b,c), escalating dosages of pre‐treated sRBCs yielded a discernible reduction in liver accumulation, thus augmentation in spleen accumulation for subsequently injected ZAlum/OVA nanovaccines. Specifically, the spleen/liver accumulation ratio is ∼0.89 in the absence of sRBCs pre‐treatment, and escalating sRBCs dosages favor a higher spleen/liver accumulation ratio (∼2.29 for pre‐treatment using 3 D sRBCs) (Figure 2d). Furthermore, the quantification of ZAlum/OVA nanovaccines accumulation within the spleen as measured by ICP‐MS, reveals an impressive 0.8‐fold augmentation in spleen accumulation due to the sRBCs pre‐treatment (Figure 2e).

Intrigued by the tumor accumulation of ZAlum/OVA nanovaccines, we performed a time‐course in vivo fluorescence imaging of tumor‐bearing mice at 8, 16, 20 and 24 h after intravenous (i.v.) injection of Cy5‐ZAlum/OVA nanovaccines with or without sRBCs pre‐injection (Figure S5, Supporting Information). Assessment of tumors collected 24 h post‐vaccination confirms that the group pre‐treated with sRBCs exhibited ≈ 1.43 times higher tumor accumulation compared with the untreated group (Figure 2f,g). Meanwhile, the mean percent injected dose (% ID) per g within the tumors reaches 3.83% in the pre‐treated group as measured by ICP‐MS, which is in line with the ex vivo fluorescence result (Figure 2h). Furthermore, the fluorescence intensities of major organs (heart, lung, kidney, spleen, and liver) show that pre‐treatment with sRBCs reduces the accumulation of ZAlum/OVA nanovaccines in the livers, kidneys and lungs (Figure S6, Supporting Information). These collective results indicate the potential of the erythrocyte‐assisted approach in orchestrating the dual accumulation of nanovaccines in both the spleen and tumor.

### 2.3 Augmented Cross‐Antigen Presentation by ZAlum Adjuvant Through Autophagy Induction

**Figure 3:**
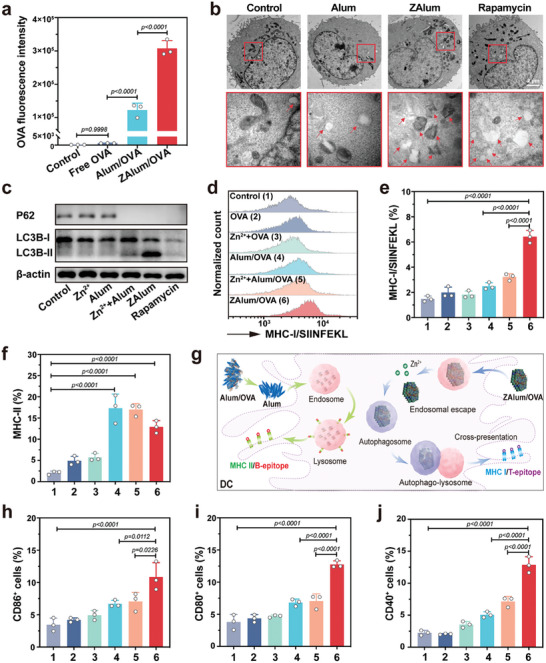
ZAlum adjuvant enhances MHC‐I antigen presentation of DCs by inducing the autophagy pathway. a) Flow cytometry analysis of antigen uptake efficiency. b) Bio‐TEM images of DC2.4 cells following different treatments for 24 h. Vacuoles following autophagy are denoted by red arrows. Scale bar = 2 µm. c) Western blot analysis of LC3B‐I/II and P62 protein in DCs with different treatments. Rapamycin was used as a positive control. d‐j) DC2.4 cells were subjected to the following treatments: 1, control; 2, OVA; 3, Zn^2+^+OVA; 4, Alum/OVA; 5, Zn^2+^+ Alum/OVA; 6, ZAlum/OVA. d‐f) The expression of OVA‐specific MHC‐I presentation (d,e) and MHC‐II presentation (f) of DCs. g) Schematic illustration of ZAlum adjuvant enhances cross‐antigen presentation via the autophagy pathway. h‐j) The expression of CD86 (h), CD80 (i), and CD40 (j) on DC2.4 cells with different treatments. Data are presented as the means ± s.d. (*n* = 3). Statistical significance was tested by one‐way ANOVA with Tukey's post hoc test.

A pivotal determinant of CD8^+^ T cell activation is the robust cross‐antigen presentation by APCs.^[^ ^26^ ^]^ DCs are professional APCs to activate T cells by presenting antigens and providing immunomodulatory signals through cell‐cell contacts and cytokine.^[^ ^27^ ^]^ Thus, we first estimated the cytocompatibility of ZAlum adjuvant to DCs, which sets the foundation for subsequent immune responses. Both ZAlum and Alum adjuvants demonstrate favorable cytocompatibility against DC2.4 cells with half maximal inhibitory concentrations (IC_50_) of 1.66 and 1.76 mg mL^−1^, respectively (Figure S7, Supporting Information).^[^ ^17^, ^28^ ^]^ Confocal images substantiate a more robust uptake of ZAlum compared to Alum, which is likely attributed to the small uniform size and conspicuous hydrophobicity of ZAlum adjuvant (Figure S8, Supporting Information). The phagocytosis of ZAlum adjuvants by DC2.4 cells facilitates the delivery of OVA, indicating the potential of ZAlum adjuvant to mediate the intracellular transfer of antigens for cellular responses (Figure S9, Supporting Information).^[^ ^23b^ ^]^ Notably, the uptake of ZAlum adjuvant remarkably boosts the internalization of the antigens by 250%, in contrast to that with Alum (**Figure** **3**a). These results indicate the capability of ZAlum adjuvant to mediate the intracellular transfer of antigens for cellular responses.

We postulated that ZAlum adjuvant might activate autophagy by releasing Zn^2+^, thereby facilitating antigen presentation in DCs. To substantiate this hypothesis, the intracellular fate of ZAlum adjuvant was investigated. During the initial stage (0.5 h), ZAlum adjuvant is internalized and transported through early endosomes. After 4 h of co‐incubation, ZAlum adjuvant escapes from lysosomes as the red fluorescence of lysosomes becomes weak,^[^ ^29^ ^]^ and at 24 h post‐incubation, a notable resurgence in lysosomal red fluorescence signifies lysosomal recovery (Figure S10, Supporting Information). The autophagy levels were detected by bio‐TEM analysis, ZAlum adjuvant shows similar autophagy‐inducing effects as Rapamycin (Figure 3b). Furthermore, a comparative analysis of ZAlum‐treated DCs against other groups in the expression of autophagy markers, specifically LC3B and P62,^[^ ^30^ ^]^ reveals a substantial elevation in the level of LC3B‐II/I, concurrently accompanied by a pronounced reduction in P62 levels (Figure 3c). The results show that ZAlum adjuvant induces a higher autophagy than Zn^2+^, Alum alone, or a mix of Zn^2+^ and Alum. Collectively, our results affirm the postulation that ZAlum adjuvant escapes from lysosomes and releases Zn^2+^ to induce autophagy.

The activation of autophagy within DCs has been noted to facilitate the processing and presentation of antigens.^[^ ^31^ ^]^ Thus, we detected the antigen presentation capability of DCs. As shown in Figure 3d,e, the results show that ZAlum/OVA nanovaccines induce the highest levels of the K^b^‐SIINFEKL complex (MHC‐I complex specific to OVA^[^ ^32^ ^]^), thereby attesting to the robust cross‐antigen presentation capability of ZAlum/OVA nanovaccines for the activation of CD8^+^ T cells. Conversely, Alum/OVA nanovaccines exhibit an elevated MHC‐II presentation capability for CD4^+^ T helper cell activation (Figure 3f; Figure S11, Supporting Information).^[^ ^33^ ^]^ Overall, Alum adjuvant seldom enters DCs, delivering antigens in its soluble form that predominantly engenders MHC‐II‐mediated antigen presentation.^[^ ^23^ ^]^ On the contrary, ZAlum/OVA nanovaccines phagocytosed by DCs and then escaped from lysosomes, harnessing the released Zn^2+^ to trigger autophagy, consequently heightening cross‐antigen presentation (Figure 3g). Importantly, antigen presentation in the absence of sufficient costimulatory signals may induce tolerance.^[^ ^27^ ^]^ Flow cytometry analysis illustrates an upregulation of various stimulatory markers (CD86, CD80, and CD40) on DCs upon incubation with ZAlum/OVA nanovaccines (Figure 3h‐j; Figure S12, Supporting Information), indicating the high adjuvanticity of ZAlum adjuvant. Collectively, ZAlum/OVA nanovaccines with profound vaccine efficacy have potential to induce robust T‐cell responses for cancer metalloimmunotherapy.

### 2.4 ZAlum/OVA Nanovaccines Trigger Cell Apoptosis and Induce ICD

**Figure 4:**
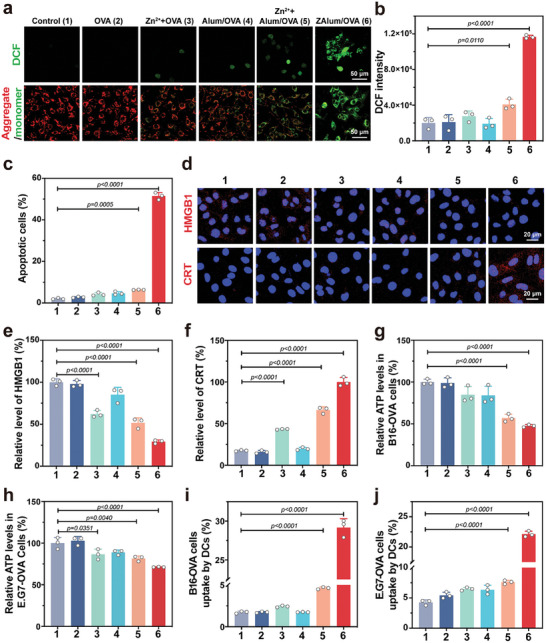
ZAlum/OVA nanovaccines trigger cell apoptosis and induce ICD in vitro. Tumor cells were subsequently treated as follows: 1, control; 2, OVA; 3, Zn^2+^+OVA; 4, Alum/OVA; 5, Zn^2+^+ Alum/OVA; 6, ZAlum/OVA. a) CLSM images (from up to down) of intracellular ROS level (DCFH‐DA staining) and mitochondrial membrane potential (JC‐1 staining) in B16F10‐OVA cells after incubating with indicated treatments. The increased JC‐1 monomer signal (green) denotes the decrease of mitochondrial membrane potential. Scale bar = 50 µm. b) ROS level in E.G7‐OVA cells with indicated treatments for flow cytometry analysis. c) The cell apoptosis study of B16F10‐OVA tumor cells after 24 h by Annevix‐V/PI kit. d) CLSM images (from up to down) of intracellular HMGB1 and CRT distribution in B16F10‐OVA cells treated with indicated treatments. Scale bar = 20 µm. e,f) Quantification of relative HMGB1 (e) and CRT (f) levels in (d). g,h) Quantitative analysis of the intracellular ATP level in B16F10‐OVA cells (g) and E.G7‐OVA cells (h) after different treatments. i,j) Phagocytosis of dying B16F10‐OVA cells (i) and E.G7‐OVA cells (j) by DC2.4 cells. Data are presented as the means ± s.d. (*n* = 3). Statistical significance was tested by one‐way ANOVA with Tukey's post hoc test.

To assess the impact of ZAlum/OVA nanovaccines on tumor cells, we initially investigated the cellular uptake of ZAlum and Alum adjuvants in vitro. Similar to their internalization by DC2.4 cells, ZAlum adjuvant was found to be taken up by B16F10‐OVA cells, whereas Alum seldom enters cells (Figure S13, Supporting Information). Thus, ZAlum adjuvant demonstrates significantly enhanced cytotoxicity against tumor cells compared to Alum. The IC_50_ values of ZAlum adjuvant against B16‐OVA and E.G7‐OVA cells are 0.81 and 0.82 mg mL^−1^, respectively (Figure S14, Supporting Information). The varying cytotoxicity of the ZAlum adjuvant against DC2.4 and tumor cells can be attributed to the inherent phagocytosis mechanism of APCs.^[^ ^17a^ ^]^ We further studied the mechanism behind the tumor cell death. Zn^2+^ has the ability to provoke reactive oxygen species (ROS) generation, which contributes to cell death.^[^ ^16a^ ^]^ We observe robust ROS generation in B16F10‐OVA and E.G7‐OVA cells treated with ZAlum/OVA nanovaccines, using 2′,7′dichlorofluorescein diacetate (DCFH‐DA) as a ROS probe (**Figure** **4**a,b; Figure S15, Supporting Information). Furthermore, excessive ROS induces mitochondrial damage.^[^ ^17a^ ^]^ CLSM images further reveal mitochondrial damage (measured by decreased mitochondrial membrane potential using JC‐1) in ZAlum/OVA‐treated B16‐OVA cells (Figure 4a), contributing to a noticeable increase in the proportion of apoptotic cells (Figure 4c; Figure S16, Supporting Information).^[^ ^16b^ ^]^ These findings suggest that the ZAlum/OVA nanovaccines induce ROS generation and mitochondrial impairment through Zn^2+^ release, consequently leading to cell apoptosis.

To explore the potential induction of ICD by the ZAlum/OVA nanovaccines, we investigated the release of damage‐associated molecular patterns (DAMPs), including high‐mobility group box 1 protein (HMGB1, a “find‐me” signal), adenosine triphosphate (ATP, a “find‐me” signal), and calreticulin (CRT, an “eat‐me” signal).^[^ ^34^ ^]^ As shown in Figure 4d‐f, the retention of HMGB1 in the cell nucleus is much lower and the exposure of CRT is much higher in ZAlum/OVA nanovaccines treated group compared to other groups. Additionally, the intracellular ATP levels decreased by 47.57% in B16F10‐OVA cells and 71.29% in E.G7‐OVA cells upon treatment with ZAlum/OVA nanovaccines (Figure 4g,h). Moreover, tumor cells exposed to ZAlum/OVA nanovaccines exhibit enhanced phagocytosis by DC2.4 cells (Figure 4i,j; Figure S17, Supporting Information). Collectively, the ZAlum/OVA nanovaccine‐mediated ICD promotes DAMPs release and tumor cell phagocytosis by DCs, providing the basis for the activation of iTAA‐specific T cells.

### 2.5 Therapeutic Effects on Melanoma and Lymphoma Mice

**Figure 5:**
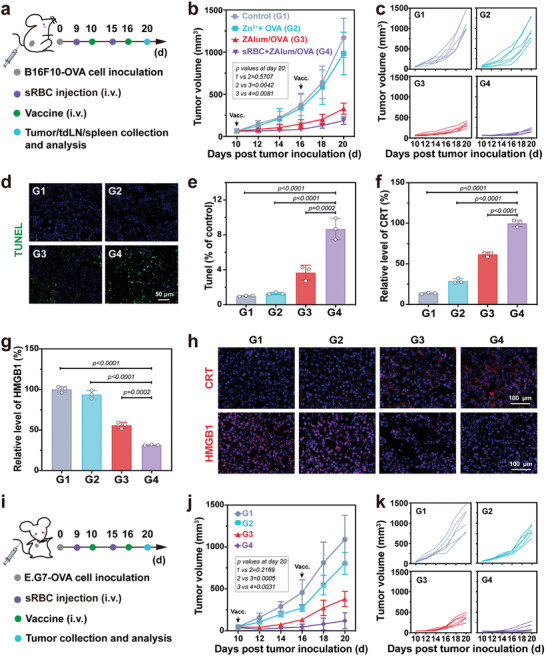
Tumor therapeutic efficacy on melanoma and lymphoma mice. B16F10‐OVA and E.G7‐OVA tumor‐bearing mice were subsequently treated as follows: G1, control; G2, Zn^2+^+OVA; G3, ZAlum/OVA; G4, sRBC+ZAlum/OVA. a) Schematic illustrating the therapeutic procedure on the melanoma mice. b,c) The average (b) and individual (c) volume of the melanoma tumor with indicated treatments (*n* = 6). d) Representative TUNEL staining of melanoma tumors. Scale bar = 50 µm. e) Quantification of TUNEL‐positive cells (*n* = 3). f,g) Quantification of relative CRT (f) and HMGB1 (g) levels in (h) (*n* = 3). h) CRT exposure and HMGB1 release in tumor tissue from B16F10‐OVA tumor‐bearing mice with indicated treatments. Scale bar = 100 µm. i) Schematic illustrating the therapeutic procedure on the lymphoma mice. sj,k) The average (j) and individual (k) volume of the lymphoma tumor with indicated treatments (*n* = 6). Data are presented as the means ± s.d. Statistical significance was tested by one‐way ANOVA with Tukey's post hoc test.

To evaluate the therapeutic efficiency of erythrocyte‐assisted systemic immunization with ZAlum/OVA nanovaccines, we conducted experiments on both B16F10‐OVA and E.G7‐OVA tumor models. The therapeutic procedure on the melanoma mice is shown in **Figure** **5**a and Figure S18a, Supporting Information. Specifically, the mice were treated with different nanovaccine formulations, and the sRBC+ZAlum/OVA group received i.v. injection of sRBCs 16 h in advance. The intratumorally (i.t.) administration of Alum/OVA and Zn^2+^+Alum/OVA nanovaccines resulted in moderate inhibition rates of ≈19% and 27%, respectively. Notably, the i.t. administration of ZAlum/OVA nanovaccines demonstrates a substantially higher tumor inhibition rate of ≈54% (Figure S18b,c and S19, Supporting Information). Furthermore, the i.v. administration of ZAlum/OVA nanovaccines exhibits an 18% increase in tumor inhibition rate compared to the i.t. administration. Impressively, the sRBC+ZAlum/OVA treated group (≈84%) displays an enhanced tumor inhibition rate compared to the ZAlum/OVA group (≈72%) (Figure 5b,c; Figure S19, Supporting Information), which was further validated by terminal deoxynucleotidyl transferase dUTP nick‐end labeling (TUNEL) staining (Figure 5d,e). Moreover, the exposure of CRT and release of HMGB1 in tumor tissues demonstrates a significant increase in the sRBC+ZAlum/OVA treated group compared to the ZAlum/OVA group (Figure 5f‐h). Consistently, in the lymphoma mice model, the sRBC+ZAlum/OVA group exhibited an increased tumor inhibition rate of ≈23% compared to the ZAlum/OVA group (Figure 5i‐k). Collectively, these results suggest the substantial tumor growth suppression achieved through the synergistic approach of sRBC+ZAlum/OVA.

Furthermore, the biosafety of the immunization strategy was thoroughly evaluated. The body weight of mice shows a slow growth trend (Figure S20a, Supporting Information), and the levels of hepatic function markers (alanine transaminase and aspartate aminotransferase) and renal function markers (creatinine and blood urea nitrogen) in serum were observed without significant change in the group with different treatments (Figure S20b‐e, Supporting Information). Moreover, histological examination of major organs (heart, liver, spleen, lung, and kidney) displays no notable histologic abnormalities in mice treated with the sRBC+ZAlum/OVA approach (Figure S20f, Supporting Information), demonstrating the favorable biological safety of this synergistic approach.

### 2.6 Cascade Activation of Antitumor Immunity

**Figure 6:**
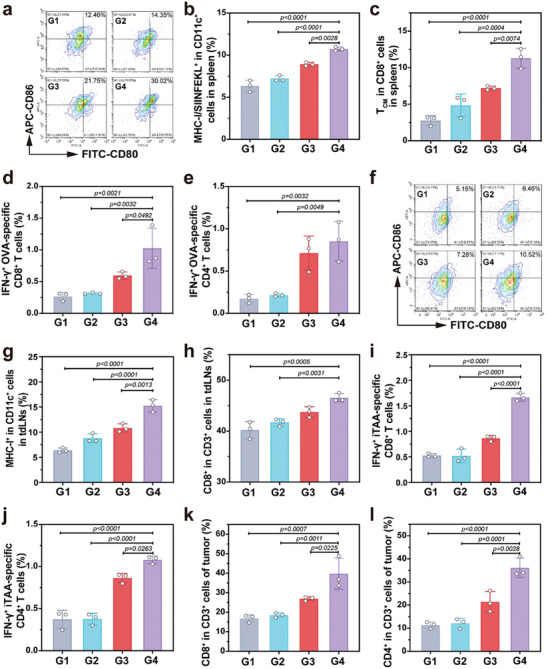
ZAlum/OVA nanovaccines mediate OVA‐specific T‐cell immunity and boost iTAA‐specific T‐cell immunity via ICD induction. B16F10‐OVA tumor‐bearing mice were subsequently treated as follows: G1, control; G2, Zn^2+^+OVA; G3, ZAlum/OVA; G4, sRBC+ZAlum/OVA. a‐c) Levels of mature DCs (CD80^+^ CD86^+^) (a), CD11c^+^ MHC‐I/SIINFEKL^+^ cells (b) and T_CM_ cells (c) in the spleen for flow cytometry analysis. d,e) The level of OVA‐specific CD8^+^ IFN‐γ^+^ T cells (d) and OVA‐specific CD4^+^ IFN‐γ^+^ T cells (e) in splenocytes after restimulation with OVA for flow cytometry analysis. f‐h) Levels of mature DCs (f), CD11c^+^ MHC‐I^+^ cells (g) and CD3^+^ CD8^+^ T cells (h) in tdLNs for flow cytometry analysis. i‐j) Levels of iTAA‐specific CD8^+^ IFN‐γ^+^ T cells (i) and CD4^+^ IFN‐γ^+^ T cells (j) after restimulation with iTAAs for flow cytometry analysis. k,l) Levels of CD3^+^ CD8^+^ T cells (k) and CD3^+^ CD4^+^ T cells (l) in tumors for flow cytometry analysis. Data are presented as the mean ± s.d. (*n* = 3). Statistical significance was tested by one‐way ANOVA with Tukey's post hoc test.

The activation of antitumor immunity was assessed in B16F10‐OVA tumor‐bearing mice, using different nanovaccine formulations on days 10 and 16 twice. Encouraged by the high adjuvanticity of ZAlum adjuvant, spleen‐specific immunization was further investigated in vivo. As expected, both ZAlum/OVA and sRBC+ZAlum/OVA treated groups exhibit pronounced autophagy activation in the spleen (Figure S21, Supporting Information). The sRBC+ZAlum/OVA group displays even greater enhancement due to the elevated spleen accumulation of ZAlum/OVA nanovaccines. Subsequently, single‐cell suspensions were obtained from spleens for flow cytometry analysis after tissue digestion. The sRBC+ZAlum/OVA group demonstrates a higher proportion of mature DCs (gated by CD11c^+^ CD80^+^ CD86^+^) (**Figure** **6**a; Figure S22, Supporting Information), along with elevated levels of SIINFEKL peptide presented on the surface of DCs (Figure 6b; Figure S23, Supporting Information). Moreover, ZAlum/OVA and sRBC+ZAlum/OVA treated groups show a 2.61‐ and 4.09‐fold increase in the level of central memory T lymphocyte (T_CM_, gated by CD3^+^ CD8^+^ CD44^+^ CD62L^+^) compared to control, respectively (Figure 6c; Figure S24, Supporting Information). Furthermore, the splenocytes harvested from immunized mice were restimulated ex vivo with OVA to evaluate the activation of OVA‐specific T cells. An increase in the level of CD8^+^ IFN‐γ^+^ T cells by 1.72 times (Figure 6d; Figure S25a, Supporting Information) and CD4^+^ IFN‐γ^+^ T cells by 1.20 times (Figure 6e; Figure S25b, Supporting Information) was observed in the sRBC+ZAlum/OVA treated group compared to the ZAlum/OVA group. These findings collectively suggest that the erythrocyte‐assisted immunization with ZAlum/OVA nanovaccines enhances OVA‐specific T‐cell activation.

To evaluate iTAA‐specific T‐cell immunity induced by ICD, we examined subsequent immune cell activation in tumor draining lymph nodes (tdLNs). The percentage of mature DCs increases notably in the sRBCs pretreated group, promoted by the release of DAMPs from B16‐OVA cells (Figure 6f; Figure S26, Supporting Information).^[^ ^34a^ ^]^ Additionally, the percentage of CD11c^+^ MHC‐I^+^ cells in tdLNs also experience a significant increase from 10.8% (ZAlum/OVA group) to 15.3% (sRBC+ZAlum/OVA group) (Figure 6g; Figure S27, Supporting Information). Moreover, CD3^+^ CD8^+^ T cells show an increase from 40.21% (untreated mice) to 46.82% (sRBC+ZAlum/OVA) in tdLNs (Figure 6h; Figure S28, Supporting Information). Furthermore, the splenocytes harvested from immunized mice were restimulated ex vivo with iTAAs (TAAs without OVA) to evaluate the activation of iTAA‐specific T cells. An increase in the level of CD8^+^ IFN‐γ^+^ T cells by 1.93 times (Figure 6i; Figure S29a, Supporting Information) and CD4^+^ IFN‐γ^+^ T cells by 1.25 times (Figure 6j; Figure S29b, Supporting Information) was observed in the sRBC+ZAlum/OVA treated group compared to the ZAlum/OVA group. These results indicate that the erythrocyte‐assisted immunization with ZAlum/OVA nanovaccines is capable of inducing iTAA‐specific T‐cell immunity. Overall, the cascade immune activation process, which combines OVA‐ and iTAA‐specific T‐cell activation amplifies cancer metalloimmunotherapy through synergistic regulation of the spleen and TME.

Capitalizing on the cascade immune activation and ICD induction, a higher proportion of T cells (CD3^+^ CD8^+^ T cell, 39.77% versus 29.92%; CD3^+^ CD4^+^ T cell, 36.38% versus 21.4%) are observed in the tumor tissue of the sRBC+ZAlum/OVA treated group compared to the ZAlum/OVA treated group (Figure 6k,l; Figure S30, Supporting Information). Moreover, the sRBC+ZAlum/OVA treated group dramatically elevated intratumoral secretion of the proinflammatory cytokines, including interleukin‐12 and tumor necrosis factor‐α , close verifying the robust activation of antitumor immune response (Figure S31, Supporting Information). These results emphasize the potent therapeutic impact of erythrocyte‐assisted systemic immunization, where ZAlum/OVA nanovaccines accumulate in the spleen for DC maturation, antigen presentation, and activation of OVA‐specific T cells, and in the tumor tissue where they induce ICD, leading to iTAA‐specific T‐cell immunity and enhanced infiltration of T cells.

## 3 Conclusion

Cancer therapeutic vaccines have shown great promise in the field of clinical cancer immunotherapy. However, their therapeutic efficacy is significantly impeded by the slow onset of immune responses and the challenge of eliciting robust immune reactions. In this study, we introduce a cascade immune activation paradigm by employing erythrocyte‐assisted systemic immunization with immunomodulatory ZAlum/OVA nanovaccines. This approach orchestrates rapid and potent cancer metalloimmunotherapy through coordinated modulation of both the spleen and the TME. Specifically, the strategic injection of sRBCs neutralizes liver macrophages, facilitating the dual accumulation of ZAlum/OVA nanovaccines in both the spleen and the TME. In the spleen, ZAlum/OVA nanovaccines play a pivotal role in activating OVA‐specific T cells through autophagy‐dependent antigen presentation in DCs. Simultaneously, within the TME, ZAlum/OVA nanovaccines leverage ICD to induce activation of iTAA‐specific T cells and enhance T cell infiltration. Such a synergistic approach demonstrated remarkable therapeutic efficacy in inhibiting tumor growth in both B16F10‐OVA and E.G7‐OVA models. Indeed, recent evidence has suggested that diverse biomaterials, such as MXene and mesoporous silica, among others,^[^ ^35^ ^]^ can activate DC‐mediated immune responses. Combining these immunization strategies with a range of biomaterials is expected to effectively enhance potent cancer immunotherapy.

In summary, we have developed an erythrocyte‐assisted systemic immunization strategy with ZAlum/OVA nanovaccines, which harnesses spatial immunomodulation between the spleen and the TME. This approach amplifies antitumor immunity through cascade immune activation and increased T cell infiltration, significantly enhancing tumor suppression with minimal side toxicity. Additionally, the facile preparation and excellent stability of ZAlum/OVA nanovaccines make this strategy highly promising for clinical translation. While conventional cancer therapeutic vaccines aim to direct the immune system to eliminate cancer cells, they often face limitations due to the immunologically cold TME. In contrast, our approach not only rapidly induces vaccine antigen‐specific T cell immunity in the spleen but also triggers potent in situ antigen‐specific T cell immunity through tumor ICD induction, such a collaborative spatial immunization effectively connects the spleen and the TME. Consequently, the erythrocyte‐assisted cascade immune activation using ZAlum/OVA nanovaccines results in rapid and robust antitumor immunity induction, holding great potential for clinical cancer metalloimmunotherapy.

## Conflict of Interest

The authors declare no conflict of interest.

## Supporting information

Supporting Information

## Data Availability

The data that support the findings of this study are available from the corresponding author upon reasonable request.
