# Supplementary material for: An Immunomodulatory Zinc‐Alum/Ovalbumin Nanovaccine Boosts Cancer Metalloimmunotherapy Through Erythrocyte‐Assisted Cascade Immune Activation
Source: Adv Sci (Weinh). 2023 Dec 8;11(6):2307389. doi: 10.1002/advs.202307389 (PMC10853754; doi:10.1002/advs.202307389)
Supplement: Supplementary file 1 — Supporting Information [file ADVS-11-2307389-s001.pdf]

ADVANCED SCIENCE
Open Access

Supporting Information

for *Adv. Sci.*, DOI 10.1002/advs.202307389

An Immunomodulatory Zinc-Alum/Ovalbumin Nanovaccine Boosts Cancer Metalloimmunotherapy Through Erythrocyte-Assisted Cascade Immune Activation

Jing Zhao, Lingxiao Zhang, Pin Li, Shanbiao Liu, Shiyi Yu, Zheng Chen, Mingjian Zhu, Shangzhi Xie, Daishun Ling and Fangyuan Li**

Supporting Information

An Immunomodulatory Zinc-Alum/Ovalbumin Nanovaccine Boosts Cancer Metalloimmunotherapy through Erythrocyte-Assisted Cascade Immune Activation

Jing Zhao[#], Lingxiao Zhang[#], Pin Li, Shanbiao Liu, Shiyi Yu, Zheng Chen, Mingjian Zhu, Shangzhi Xie, Daishun Ling, Fangyuan Li**

J. Zhao, P. Li, S. Liu, S. Yu, Z. Chen, M. Zhu, S. Xie, Prof. D. Ling, Prof. F. Li
Institute of Pharmaceutics, Hangzhou Institute of Innovative Medicine, College of Pharmaceutical Sciences, Zhejiang University, Hangzhou 310058, P. R. China.
E-mail: dsling@sjtu.edu.cn (D. Ling), ORCID: 0000-0002-9977-0237 (D. Ling); lfy@zju.edu.cn (F. Li)

Prof. L. Zhang
Interdisciplinary Nanoscience Center (iNANO), Aarhus University, Aarhus C DK-8000, Denmark.

Prof. D. Ling
Frontiers Science Center for Transformative Molecules, School of Chemistry and Chemical Engineering, State Key Laboratory of Oncogenes and Related Genes, National Center for Translational Medicine, Shanghai Jiao Tong University, Shanghai 200240, P. R. China.

Prof. D. Ling, Prof. F. Li
WLA Laboratories, Shanghai 201203, P. R. China.

Prof. F. Li
Key Laboratory of Precision Diagnosis and Treatment for Hepatobiliary and Pancreatic Tumor of Zhejiang Province Hangzhou 310009, P. R. China.

# These authors contributed equally to this work.

Keywords: Cancer metalloimmunotherapy, Nanovaccines, Erythrocytes, Spleen, Tumor microenvironment.

Supplementary Notes

The Optimization of ZAlum Adjuvant: Transmission electron microscope (TEM) images show that nanoparticles (NPs) synthesized at molar ratios of 1:1 and 3:1 are accompanied by heterogeneous materials (Figure S1a,b, Supporting Information). X-ray diffraction (XRD) patterns (Figure S1c, Supporting Information) and Fourier transform infrared (FT-IR) spectra (Figure S1d, Supporting Information) confirm the formation of hydrotalcite-like structure in all compounds (JCPDS No. 38-0486),[1] and reveal that the heterogeneous materials at the molar ratio of 1:1 attributed to the deposition of incompletely hydrolyzed $Zn(OH)_2$ (JCPDS No. 38-0356) [2] and the crystallization of ZnO (JCPDS No. 36-1451)[3] on zinc hydroxide layers, which result in the high Zn/Al molar ratio (~ 3.92) measured by inductively coupled plasma mass spectrometry (ICP-MS). Besides, the final Zn/Al molar ratio at the initial molar ratio of 2:1 and 3:1 in the absence of redundant $Zn(OH)_2$ is close to 2 (Table S1, Supporting Information). Moreover, hydrodynamic size and Zeta potential (Figure 1e; Figure S1e,f, Supporting Information) are accompanied by an increase in the molar ratio of Zn/Al.

Supplementary Figures

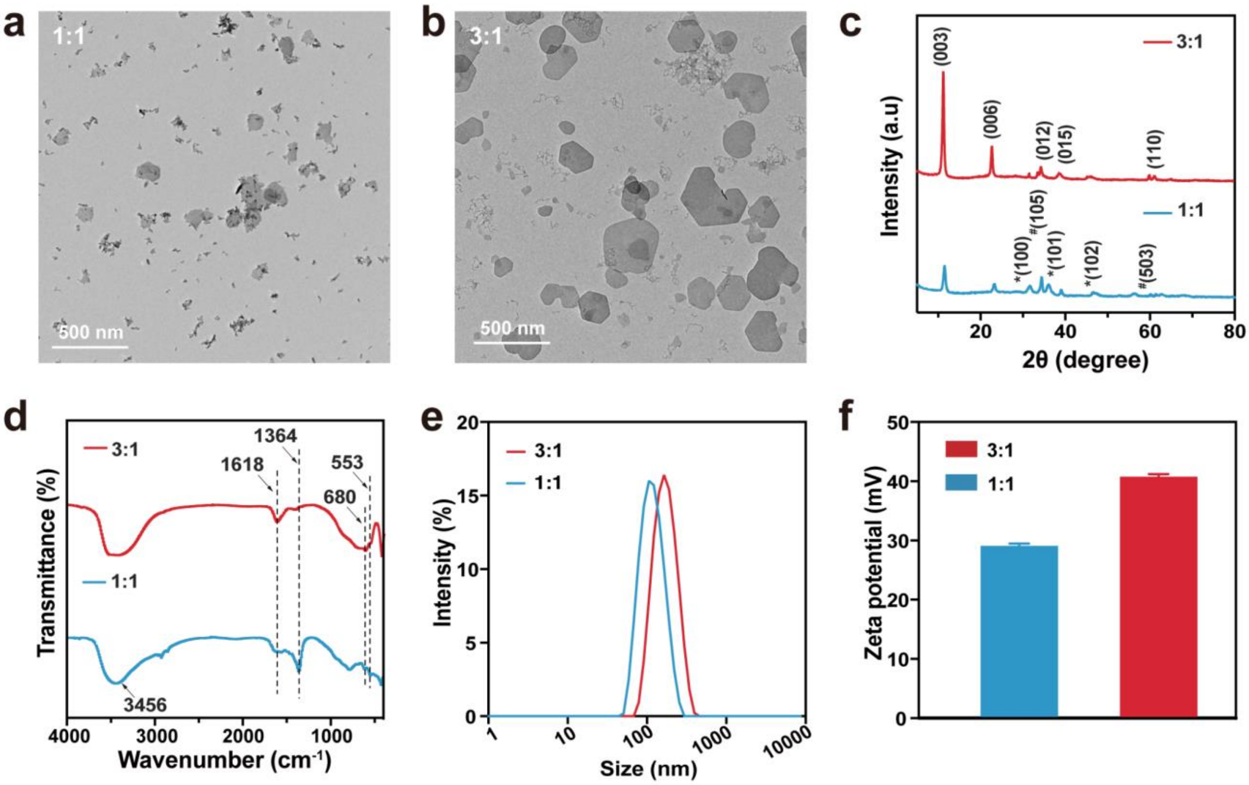

Figure S1. The optimization of ZAlum adjuvant. ZAlum was prepared by different initial Zn/Al precursor molar ratios to find the right molar ratio forming a uniform and stable adjuvant. (a,b) TEM images of NPs at initial Zn/Al precursor molar ratios of 1:1 (a) and 3:1 (b). Scale bar = 500 nm. (c) XRD patterns. Diffractions derived from ZnO (*) and $Zn(OH)_2$ ($^{\#}$) are marked. (d) FT-IR spectra. The peaks can be assigned to O-H (3456 cm^{-1} and 1618 cm^{-1}), Cl^- (1364 cm^{-1}), M-O-M (680 cm^{-1}) and M-O (553 cm^{-1}), respectively. M denotes Zn or Al. (e) Particle size distribution, (f) Zeta potential of NPs at initial Zn/Al precursor molar ratios of 1:1 (blue) and 3:1 (red). Data are presented as the mean ± s.d. (n = 3).

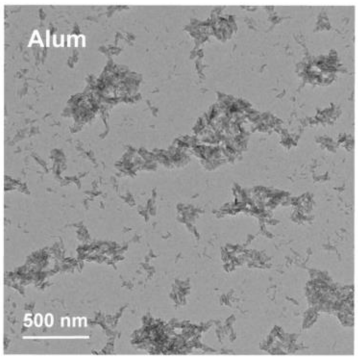

Figure S2. TEM image of Alum. Scale bar = 500 nm.

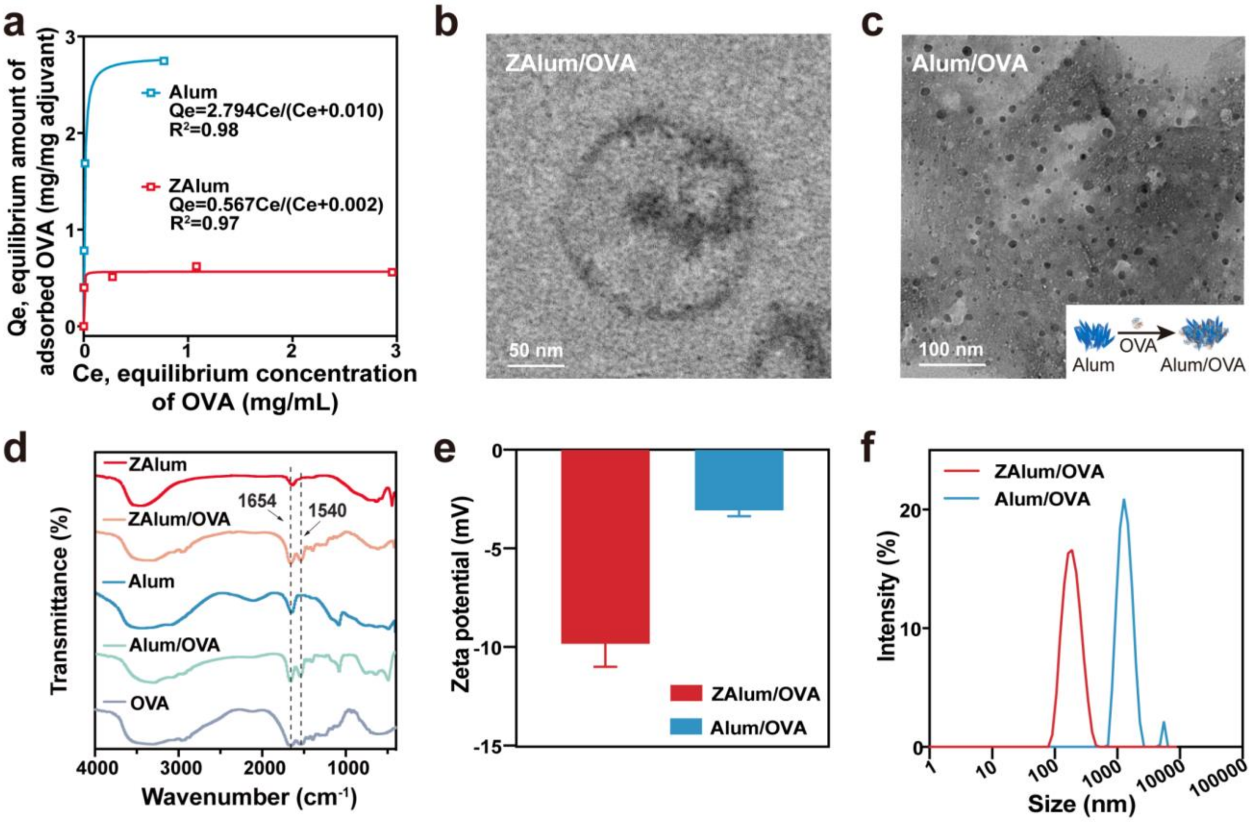

Figure S3. Preparation and characterization of ZAlum/OVA and Alum/OVA nanovaccines. (a) The OVA adsorption curve of ZAlum and Alum adjuvants. (b) TEM image of ZAlum/OVA. Scale bar = 50 nm. (c) Schematic illustration of Alum/OVA construction (insert) and TEM image of Alum/OVA. Scale bar = 100 nm. (d) FT-IR spectra of indicated NPs. The OVA characteristic peaks of C=O (1654 cm^{-1}) and N-H (1540 cm^{-1}) vibrations were observed in ZAlum/OVA and Alum/OVA. (e) Zeta potential, (f) Particle size distribution of ZAlum/OVA (red) and Alum/OVA

(blue). Data are presented as the mean ± s.d. (n = 3).

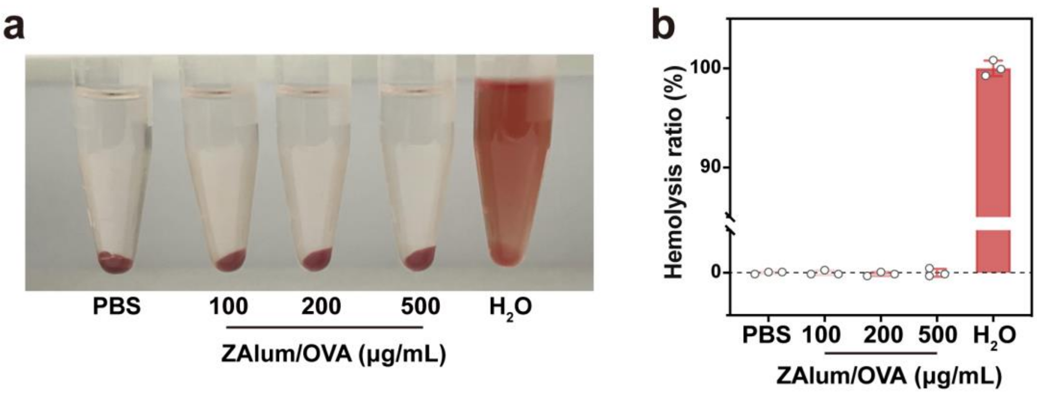

Figure S4. Representative picture (a) and hemolysis ratio (b) of fresh mouse RBCs incubated with the ZAlum/OVA nanovaccines. PBS and H_2O were used as negative and positive controls, respectively. Data are presented as the means ± s.d. (n = 3).

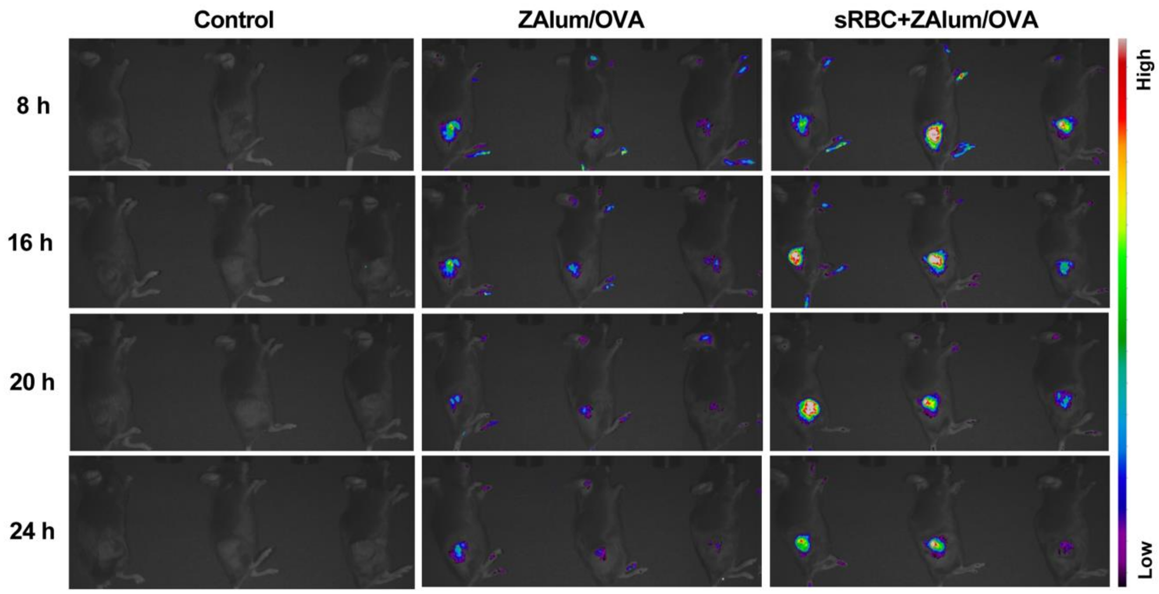

Figure S5. Representative in vivo fluorescence imaging of tumor-bearing mice at the indicated time points (8, 16, 20, and 24 h) after intravenous injection of Cy5-ZAlum/OVA nanovaccine with or without sRBCs pre-injection.

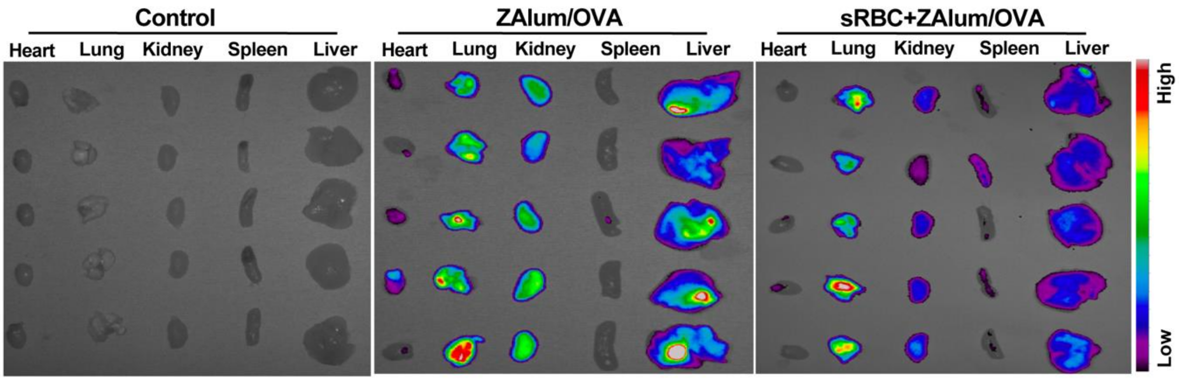

Figure S6. Representative ex vivo fluorescence images of heart, lung, kidney, spleen, and liver at 24 h after intravenous injection of Cy5-ZAlum/OVA nanovaccine with or without sRBCs pre-injection.

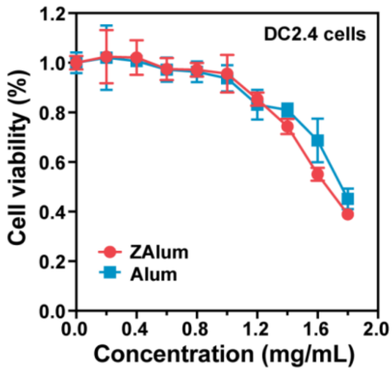

Figure S7. The cell viability of DC2.4 cells after incubating with ZAlum and Alum adjuvants for 24 h. Data are presented as the mean ± s.d. (n = 6).

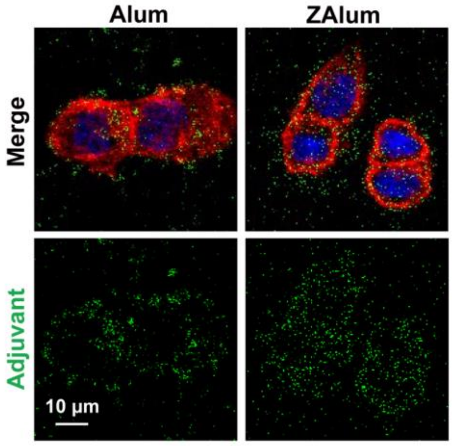

Figure S8. CLSM images of engulfment in DC2.4 cells treated with Alum or ZAlum adjuvants for 4 h. Illustrating higher uptake of ZAlum adjuvant (right), in contrast to that of Alum adjuvant (left). DC membrane and adjuvant (Alum or ZAlum) were labelled with TRITC-phalloidin (red) and Lumogallion (green), respectively. Scale bar = 10 μm.

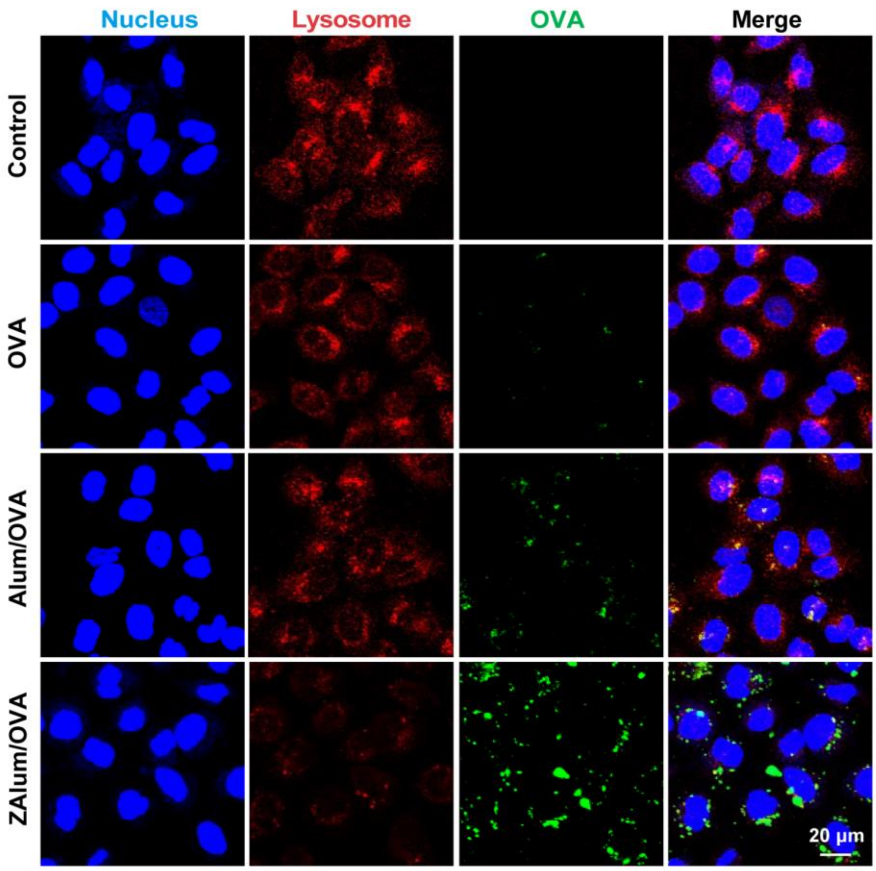

Figure S9. CLSM images of DC2.4 cells after incubated with Alum/OVA or ZAlum/OVA nanovaccine for 4 h. Alum/OVA or ZAlum/OVA nanovaccines and lysosomes were labelled by Cy5 (green) and Lysotracker Red (red), respectively. Scale bar = 20 μm.

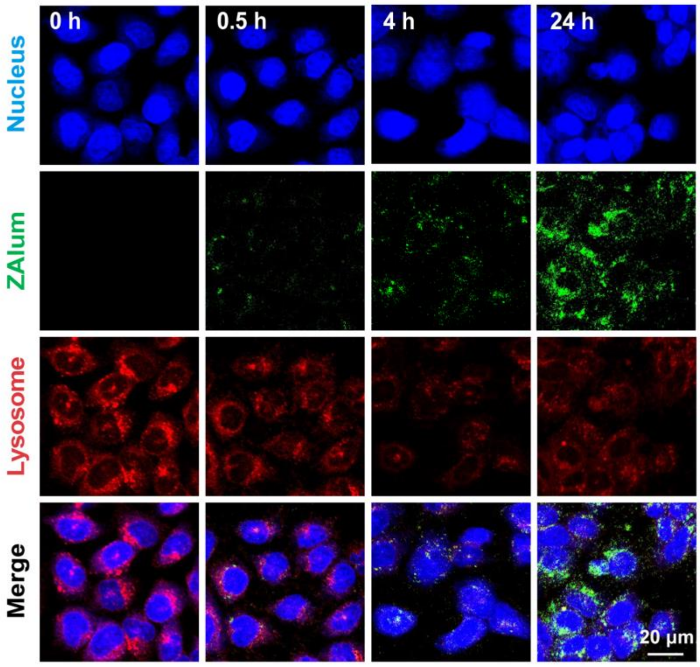

Figure S10. CLSM images of DC2.4 cells after incubation with ZAlum adjuvant for 0.5, 4 or 24 h. Adjuvants and lysosomes were labelled by Lumogallion (green) and Lysotracker Red (red), respectively. Scale bar = 20 µm.

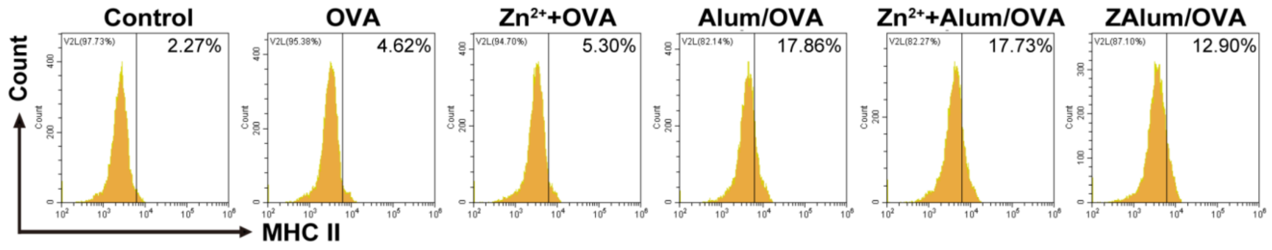

Figure S11. Effect of the ZAlum/OVA nanovaccine on the MHC-II expression of DC2.4 cells. The proportion of MHC-II^{+} cells was analyzed by flow cytometry after DC2.4 cells incubated with OVA, Zn^{2+}+OVA, Alum/OVA, Zn^{2+} + Alum/OVA or ZAlum/OVA for 24 h.

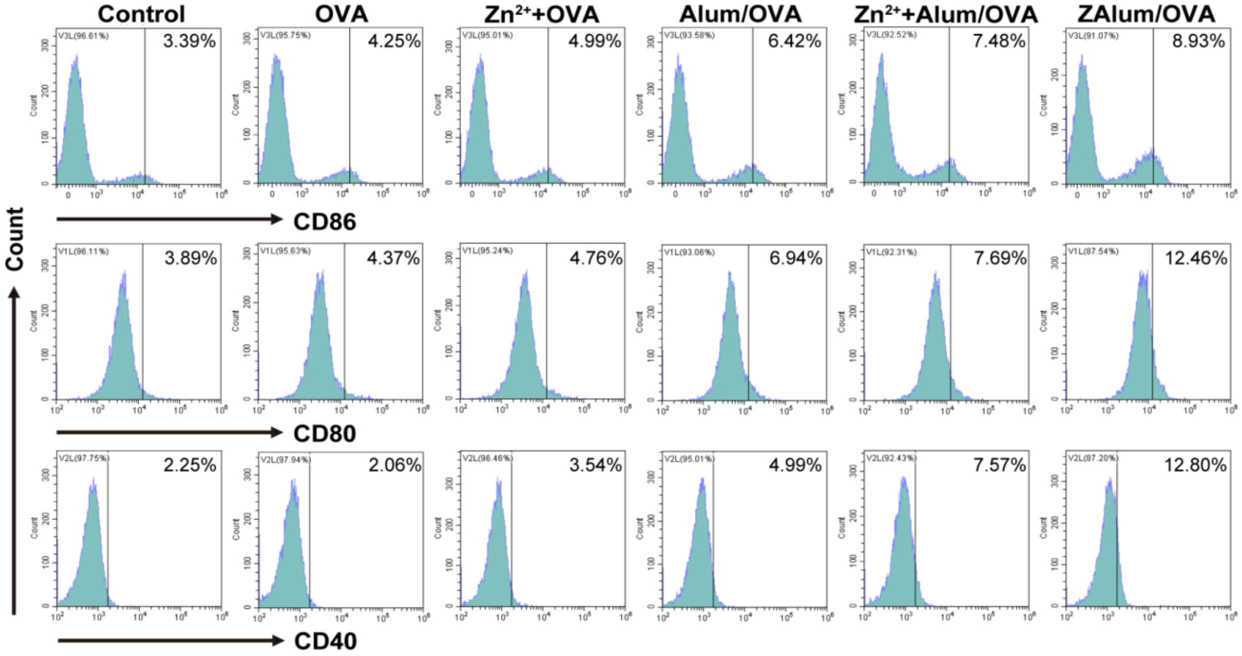

Figure S12. Effects of the ZAlum/OVA nanovaccine on the DC maturation. The proportion of CD86$^+$, CD80$^+$ or CD40$^+$ cells was analyzed by flow cytometry after DC2.4 cells incubated with OVA, Zn^{2+}+OVA, Alum/OVA, Zn^{2+} + Alum/OVA or ZAlum/OVA for 24 h.

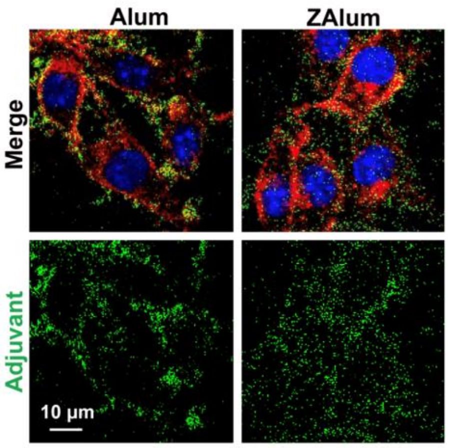

Figure S13. CLSM images of engulfment in B16F10-OVA cells treated with Alum or ZAlum adjuvant for 24 h. Illustrating higher uptake of ZAlum adjuvant (right), in contrast to that of Alum adjuvant (left). B16F10-OVA membrane and adjuvant (Alum or ZAlum) were labelled with TRITC-phalloidin (red) and Lumogallion (green), respectively. Scale bar = 10 μm.

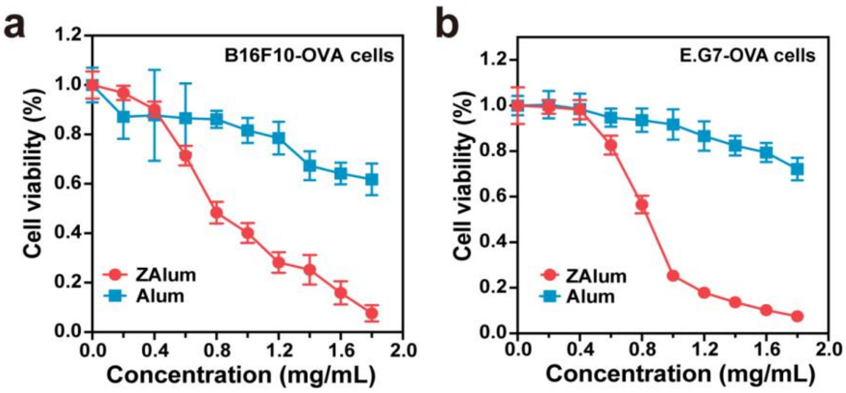

Figure S14. The cell viability of B16F10-OVA (a) and E.G7-OVA (b) tumor cells after incubating with ZAlum and Alum adjuvants for 24 h. Data are presented as the mean ± s.d. (n = 6).

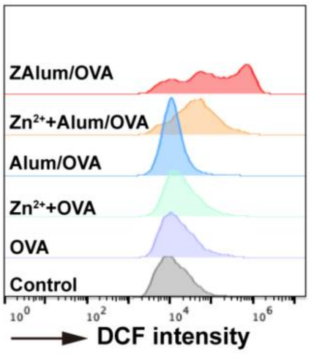

Figure S15. ROS level with indicated treatments in E.G7-OVA cells was analyzed by flow cytometry.

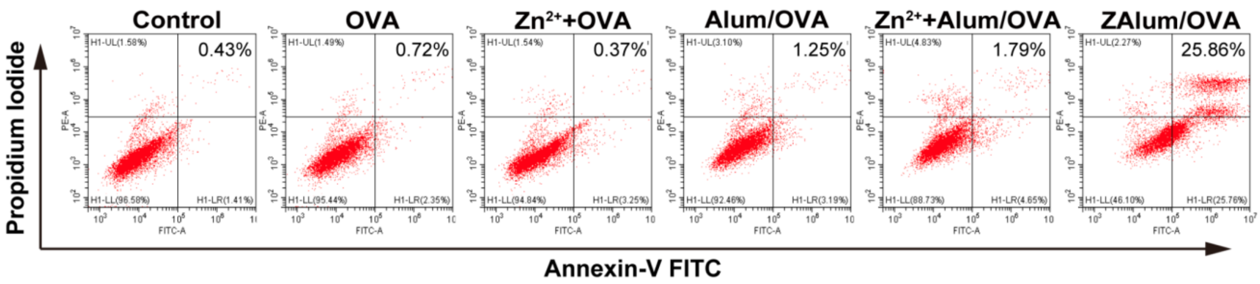

Figure S16. The cell apoptosis study of B16F10-OVA tumor cells after 24 h by Annevix-V/PI kit.

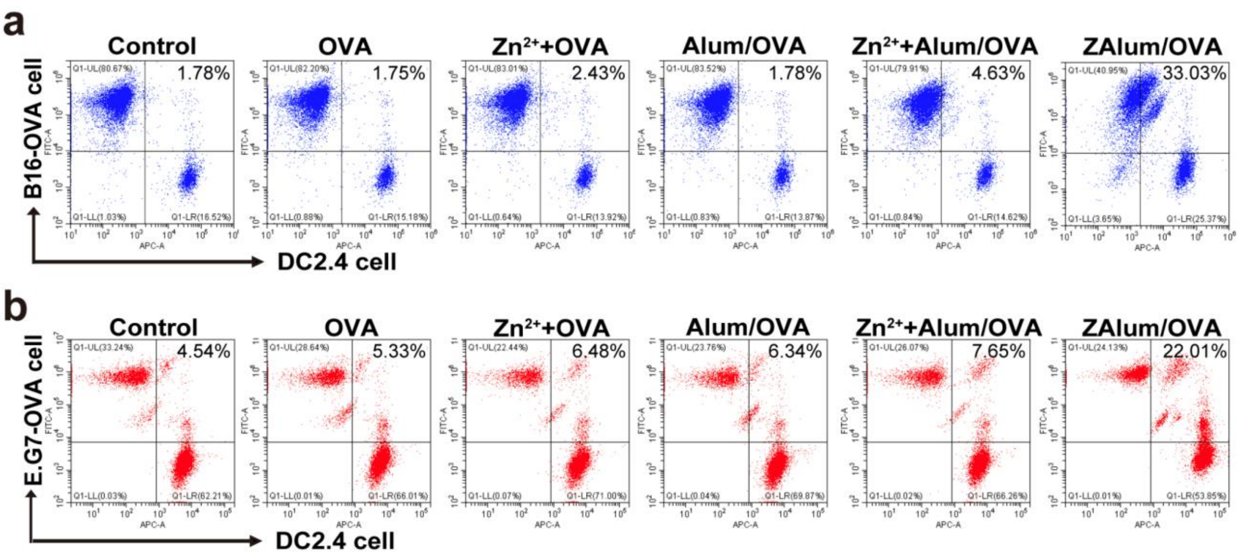

Figure S17. Phagocytosis of the dying B16F10-OVA (a) and E.G7-OVA (b) tumor cells by DC2.4 cells as analyzed by flow cytometry.

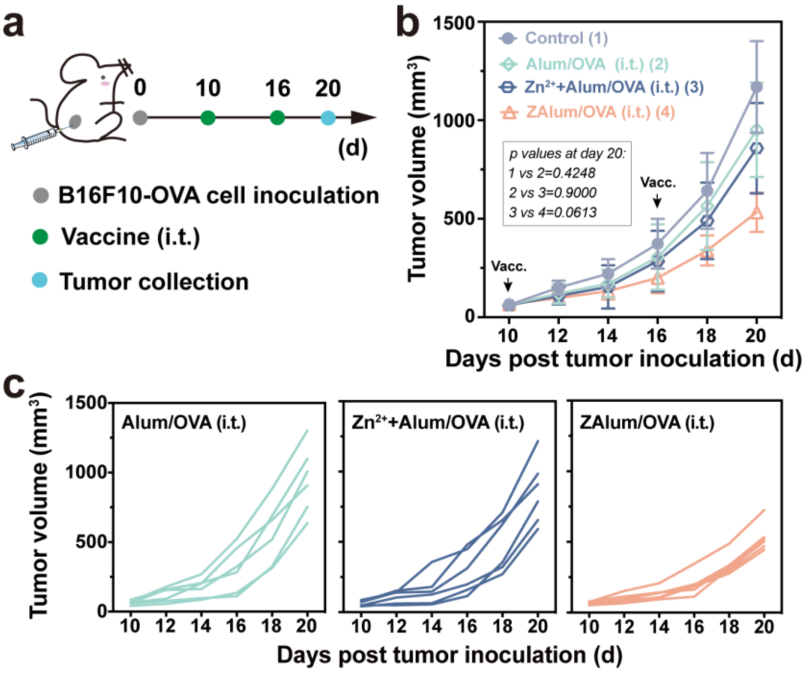

Figure S18. Tumor therapeutic efficacy on melanoma mice via intratumor (i.t.) administration. (a) Schematic illustration of the therapeutic procedure on the melanoma mice. (b,c) The average (b) and individual (c) volume of the melanoma tumor with indicated treatments (n = 6). Data are presented as the mean ± s.d.

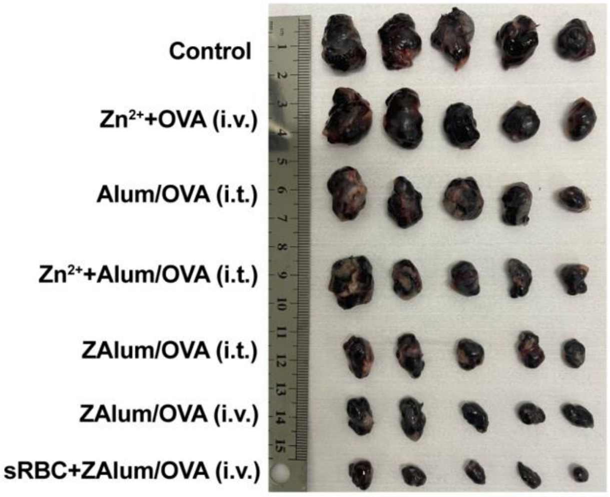

Figure S19. Optical image of melanoma tumors at day 20.

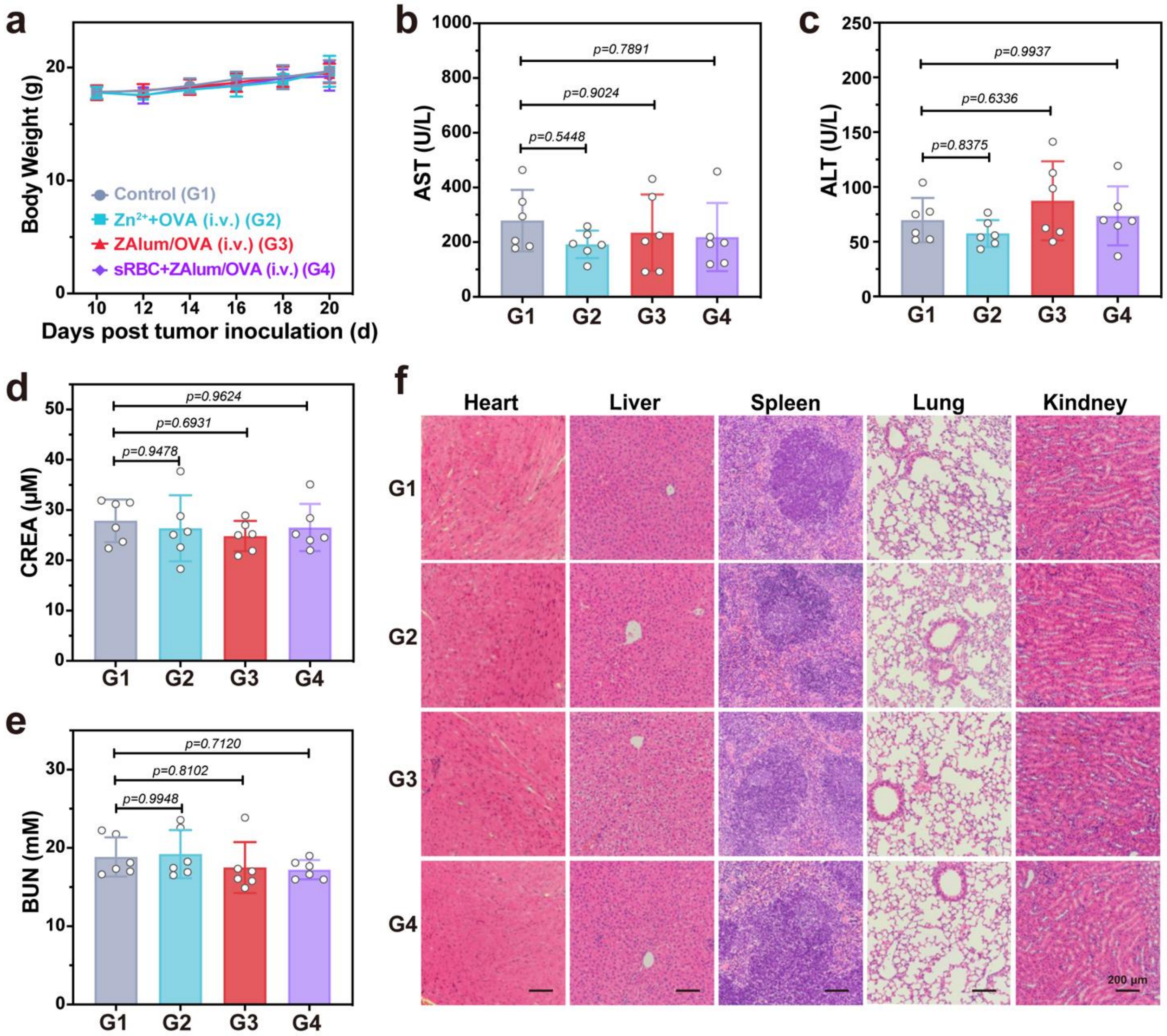

Figure S20. Biosafety analysis. Tumor-bearing mice were subsequently treated as follows: G1, control; G2, Zn^{2+}+OVA; G3, ZAlum/OVA (i.v.); G4, sRBC+ZAlum/OVA (i.v.). (a) Body weights of mice with indicated treatments (n = 6). (b-e) The levels of aspartate aminotransferase (AST) (b), alanine transaminase (ALT) (c), creatinine (CREA) (d), and blood urea nitrogen (BUN) (e) in serum of mice on the 20th day with indicated treatments (n = 6). (f) Hematoxylin and eosin (H&E) staining of main organs (heart, liver, spleen, lung, and kidney) of mice on the 20th day with indicated treatments. Scale bar = 200 μm. Data are presented as the mean ± s.d.

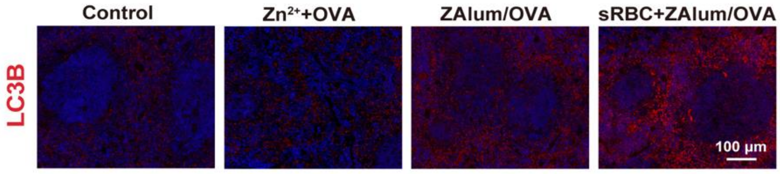

Figure S21. Representative immunofluorescence images of autophagy (LC3B, red) in the spleen. Scale bar = 100 μm.

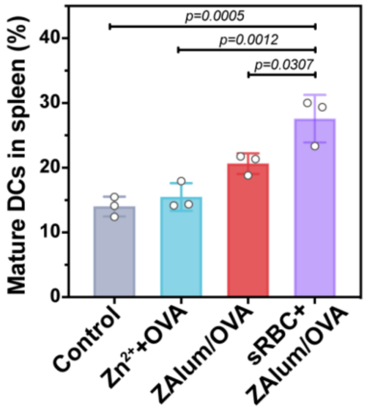

Figure S22. The level of mature DCs (CD11c$^+$ CD80$^+$ CD86$^+$) in the spleen for flow cytometry analysis.

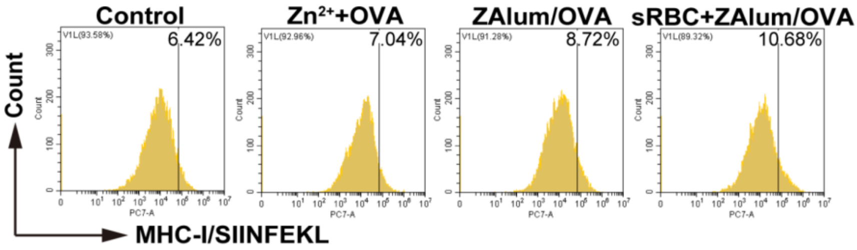

Figure S23. The level of cross-antigen presentation on DCs in the spleen. The level of CD11c$^+$ MHC-I/SIINFEKL$^+$ cells in splenocytes after systemic nanovaccine of saline, Zn^{2+}+OVA, ZAlum/OVA and sRBC+ZAlum/OVA for flow cytometry analysis.

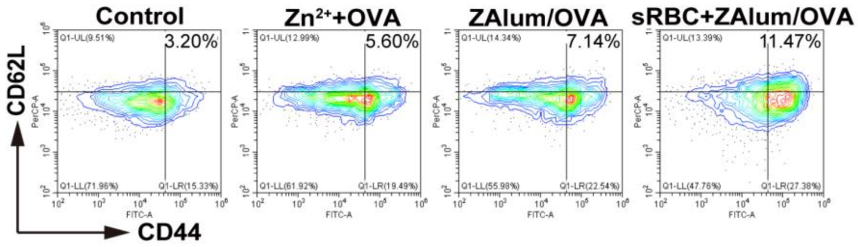

Figure S24. The level of central memory T lymphocyte (T_{CM}) in the spleen. The level of T_{CM} (gated by $CD3^+$ $CD8^+$ $CD44^+$ $CD62L^+$) cells in the spleen after systemic nanovaccine of saline, Zn^{2+}+OVA, ZAlum/OVA and sRBC+ZAlum/OVA for flow cytometry analysis.

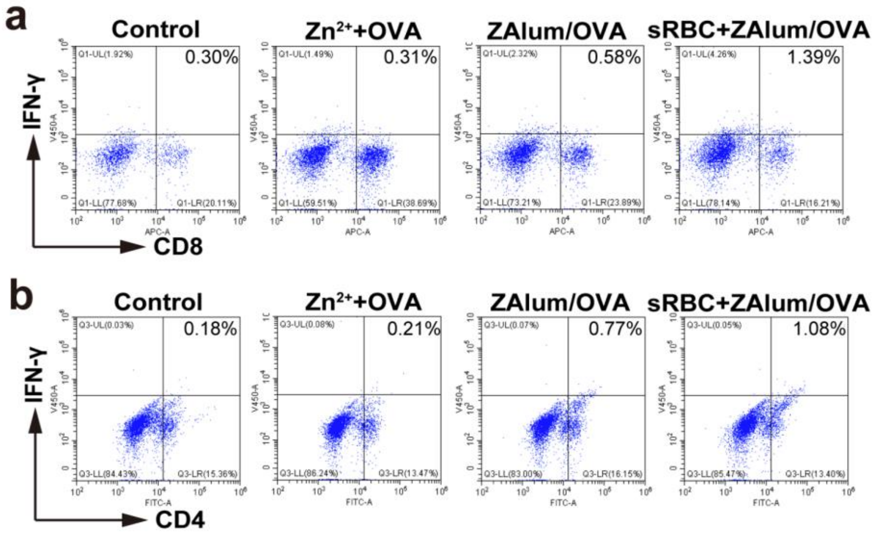

Figure S25. The levels of OVA-specific T cells in splenocytes. The levels of OVA-specific $CD8^+$ IFN-γ^+ T cells (a) and $CD4^+$ IFN-γ^+ T cells (b) in splenocytes after restimulation with OVA for flow cytometry analysis.

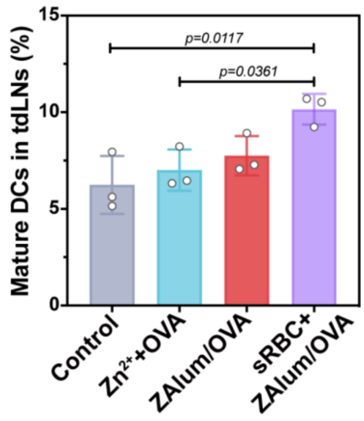

Figure S26. The level of mature DCs (CD11c^{+} CD80^{+} CD86^{+}) in the tumor draining lymph nodes (tdLNs) for flow cytometry analysis.

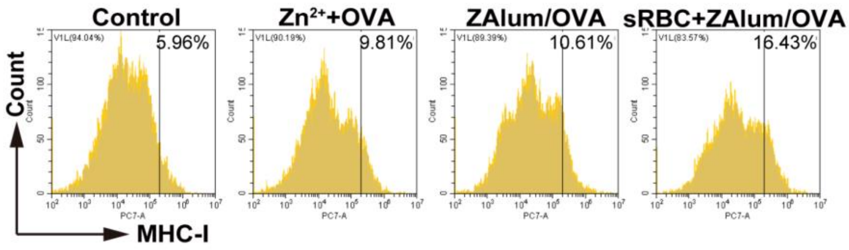

Figure S27. The level of cross-antigen presentation on DCs in the tdLNs. The level of CD11c^{+} MHC-I^{+} cells in tdLNs after systemic nanovaccine of saline, Zn^{2+}+OVA, ZAlum/OVA and sRBC+ ZAlum/OVA for flow cytometry analysis.

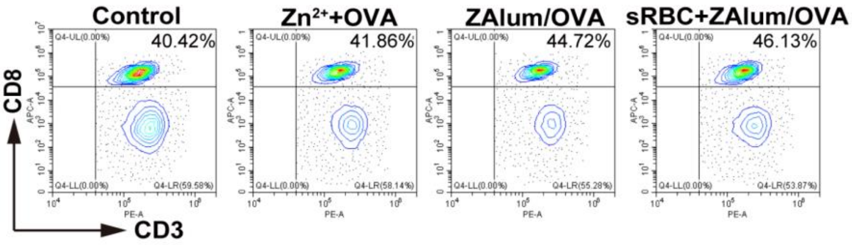

Figure S28. The level of CD3^{+} CD8^{+} T cells in tdLNs for flow cytometry analysis.

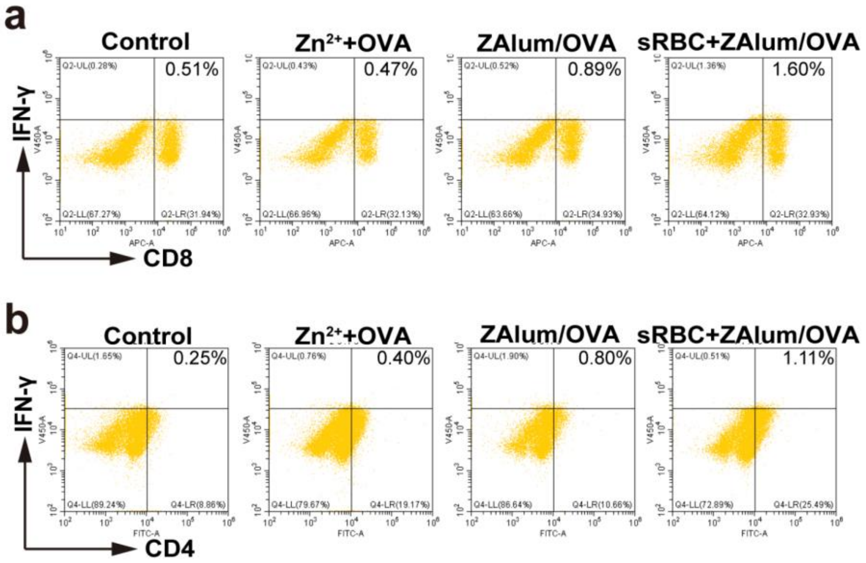

Figure S29. The levels of individualized tumor-associated antigens (iTAA)-specific T cells in splenocytes. The levels of iTAA-specific $CD8^+$ IFN-γ^+ T cells (a) and $CD4^+$ IFN-γ^+ T cells (b) in splenocytes after restimulation with iTAAs for flow cytometry analysis.

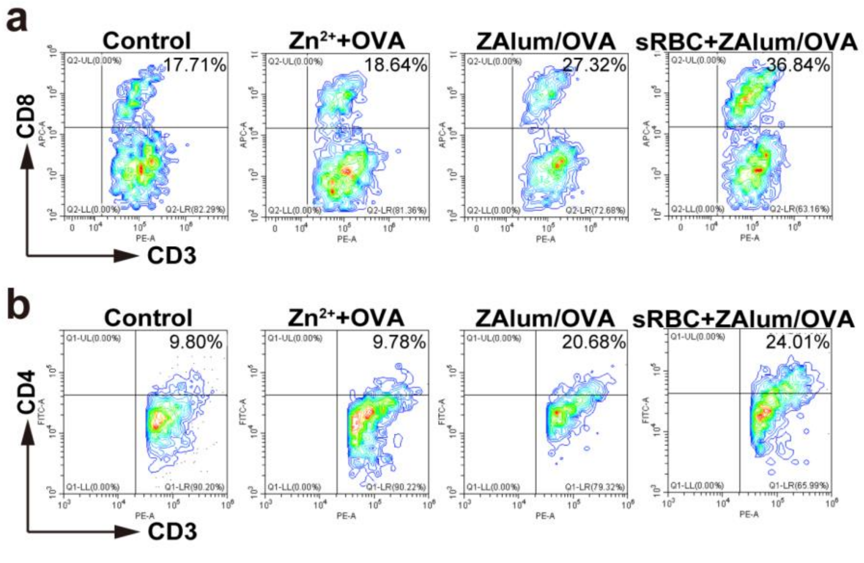

Figure S30. The levels of T cells in the tumor. The levels of $CD3^+$ $CD8^+$ T cells (a) and $CD3^+$ $CD4^+$ T cells (b) in B16F10-OVA tumor after systemic vaccine of saline, Zn^{2+}+OVA, ZAlum/OVA and sRBC+ZAlum/OVA for flow cytometry analysis.

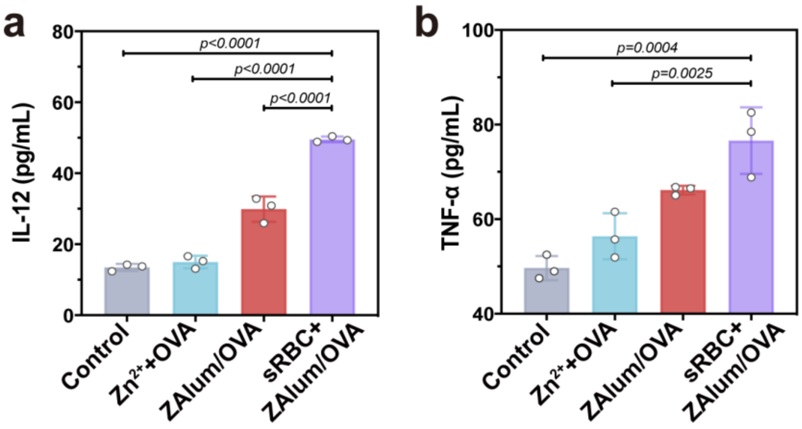

Figure S31. The ELISA assay of intratumoral secretion of (a) IL-12 and (b) TNF-α.

Supplementary Tables

Table S1. The mass and molar ratio of NPs synthesized with different initial Zn/Al precursor molar ratios.

Initial molar ratio of Zn/Al precursor	Mass ratio (Zn/Al)	Molar ratio (Zn/Al)
1:1	9.57 ± 0.55	3.92 ± 0.22
2:1 (ZAlum)	5.10 ± 0.69	2.09 ± 0.28
3:1	5.02 ± 0.57	2.05 ± 0.24

Data are presented as the mean ± s.d. (n = 3).

Table S2. The BET specific surface area and total particle surface area of ZAlum and Alum adjuvants.

Sample	BET specific surface area (m^2 g^{-1})	Total particle surface area of 1 mg of adjuvants ($x10^{10}$ μm^2)
Alum	285.7	2.77
ZAlum	27.7	28.57

Table S3. Zn^{2+} and Al^{3+} release from ZAlum/OVA (100 μg mL^{-1}) at pH 5.5, 6.5 or 7.4 after 24 h.

pH	Zn^{2+} (ng mL^{-1})	Al^{3+} (ng mL^{-1})
7.4	20.2 ± 0.6	13.8 ± 0.9
6.5	128.3 ± 1.7	31.5 ± 11.8
5.5	491.2 ± 21.0	36.3 ± 4.6

Data are presented as the mean ± s.d. (n = 3).

Supplementary Methods

Materials and Reagents: $ZnCl_2 \cdot 6H_2O$, $AlCl_3 \cdot 6H_2O$, and NaOH were purchased from Beijing Chemicals (China). Alum was purchased from Invivogen (USA). RPMI medium 1640 basic, Dulbecco's modified Eagle's medium (DMEM), fetal bovine serum (FBS) and penicillin streptomycin were purchased from Thermo Fisher Scientific (USA). Trypsin was purchased from Corning (USA). Lyso-tracker red, DAPI, Hoechst33342 and Dil were purchased from Beyotime Biotechnology (China). Lumogallion and Rapamycin were purchased from MedchemExpress (USA). Cy5-SE was purchased from Nanjing Goyoo Biotech Co., Ltd (China). 2-mercaptoethanol, G418, TRITC-phalloidin, bovine serum albumin (BSA) and ovalbumin (OVA) were purchased from Sigma-Aldrich (USA). Mouse red blood cells (RBCs) were purchased from Shanghai Yuanye Biotech Co., Ltd (China).

Cell Lines and Animals: B16F10-OVA, B16F10, E.G7-OVA, and DC2.4 cells were purchased from the China Infrastructure of Cell Line Resources (Beijing, China). B16F10-OVA and B16F10 cells cultured in DMEM culture medium containing 10% of FBS and 100 U mL^{-1} penicillin/streptomycin. E.G7-OVA and DC2.4 cells were cultured in RPMI 1640 medium containing 10% FBS, 0.05mM 2-mercaptoethanol and 0.4 mg mL^{-1} G418, and RPMI 1640 medium containing 10% FBS and 100 U mL^{-1} mixed penicillin/streptomycin, respectively. C57BL/6 female mice (6 ~ 8 weeks) were obtained from Shanghai SLAC Laboratory Animal Co., Ltd (China) and housed in a specific pathogen-free, light-cycled and temperature-controlled facility. All experiments were performed in accordance with the China Public Health Service Guide for the Care and Use of Laboratory

Animals. All animal experiment protocols were approved by the Animal Ethical Committee of Zhejiang University (SYXK (Zhejiang) 2022-0226).

Preparation of ZAlum and Other Nanoparticles: ZAlum nanoparticles (NPs) were synthesized through hydrothermal hydrolysis. In detail, 10 mL of salt mixture (molar ratio of Zn/Al = 3, 2 or 1) of $ZnCl_2$ (0.9, 0.6 or 0.3 M) and $AlCl_3$ (0.3 M) were rapidly added to 40 mL of NaOH (0.45 M) solution, and subsequently stirred vigorously for 15 min under the argon atmosphere. The slurry was collected by centrifugation and washed twice with deionized water, and then resuspended in 40 mL of deionized water and heated at 80 °C for 2 h in an oven. Finally, the product was collected by centrifugation (4000 g, 5 min) to obtain ZAlum NPs and labelled as 3:1, 2:1 (ZAlum), and1:1 respectively.

ZAlum/OVA or Alum/OVA nanovaccine was obtained according to the previous report[4]. Briefly, the OVA loading capacity of ZAlum and Alum was examined at first.1 mL of ZAlum or Alum solution (2 mg mL^{-1}) was dropwise added to 1 mL of OVA solution (0.625, 1.25, 2.5, 5 or 10 mg mL^{-1}) under ultrasonic condition for 5 min, and subsequently, the complexes were removed by centrifugation (10000 g, 10 min). Then the concentration of OVA in the supernatant was detected by a BCA kit (Epizyme, China). The maximum OVA adsorption capacity of ZAlum or Alum was estimated by the Langmuir adsorption model ($Q_e = Q_mC_e/(1/K+C_e)$, where Q_m (mg g^{-1}) is the maximum adsorption amount, Q_e (mg g^{-1}) and C_e (mg mL^{-1}) represent the equilibration amount of OVA adsorbed by ZAlum or Alum and the equilibration concentration of OVA in solution respectively, K (mL mg^{-1}) is the adsorption equilibrium constant. Next, ZAlum or Alum (2 mg mL^{-1}) was dropwise added to BSA/OVA (molar ratio = (10 Q_m-1):1) mixed solution (10 mg mL^{-1}) under ultrasonic condition for 5 min to obtain ZAlum/OVA or Alum/OVA.

To prepare Lumogallion-ZAlum or Lumogallion-Alum[5], 80 μL of Lumogallion solution (0.5 mM diluted in water) was co-incubated with 4 mg of ZAlum or Alum in 1 mL of RPMI medium on a rocking table for 12 h at RT. Then the Lumogallion-ZAlum or Lumogallion-Alum was collected by centrifugation (9000 g, 10 min) and resuspended in 1 mL RPMI medium.

To prepare Cy5-ZAlum/OVA, Cy5 was co-incubated with OVA, and then added to ZAlum or Alum solution under ultrasonic condition for 5 min, Cy5-ZAlum/OVA was collected by centrifugation (9000 g, 10 min) and resuspended in deionized water.

Characterizations: The surface morphology and structure of ZAlum, ZAlum/OVA, Alum and Alum/OVA were characterized by TEM (Hitachi HT7700, Japan). The size and Zeta potential were analyzed by dynamic light scattering (DLS; Malvern Nano ZS-90, UK). The element distribution of ZAlum was imaged by energy dispersive spectroscopy (EDS; FEI Tecnai G2 F20 S-TWIN, USA). The molar ratio of Zn/Al was detected by ICP-MS (PerkinElmer NexION 300X, USA). XRD patterns were collected using Bruker D8 ADVANCE (KS Analytical System, USA). FT-IR spectroscopy was recorded using NICOLET iS50FT-IR (Thermo Scientific, USA). The specific surface area determination was performed by Brunauer-Emmett-Teller (BET, Quantachrome Autosorb-1C, USA).

Hydrophobicity of ZAlum and Alum: The adsorption of Rose Bengal (RB, hydrophobic dye) on the ZAlum and Alum was used to determine the hydrophobicity[6]. Briefly, 20 μg mL^{-1} of RB (0.5 mL) was mixed with ZAlum or Alum (0.5 mL) with a range of concentrations (200 ~ 1200 μg mL^{-1}) in PBS (pH = 7.4, 10 mM). The control group was prepared by adding RB to PBS (0.5 mL). All the samples were mixed and incubated for 3 h and then the complexes were removed by centrifugation

(9000 g, 10 min). Next, the concentration of RB in the supernatant was detected by reading the absorbance at 542 nm (Thermo Scientific 5250040, USA). The partitioning quotient was determined by the quotient of the mass of the adsorbed RB on ZAlum or Alum and the free RB in the supernatant.

Hemocompatibility Assay: The murine blood samples were obtained from C57 mice (8 ~ 10 weeks). The 500 μL blood sample was mixed with 5 mL PBS and centrifuged (2000 rpm, 5 min) to separate the RBCs. Then, the collected RBCs were washed twice with PBS and resuspended in 5 mL PBS. Next, RBCs were mixed with PBS, water, and ZAlum/OVA nanovaccines (100 ~ 500 μg mL^{-1}), respectively. After incubation at 37°C for 3 h, the hemolytic effect was detected by reading the absorbance of the supernatant at 570 nm (Thermo Scientific 5250040, USA).

Release of Zn^{2+} and Al^{3+} from ZAlum/OVA: ZAlum/OVA (100 μg mL^{-1}) was dispersed in PBS (pH = 5.5, 6.5 and 7.4) for 24 h, then the ZAlum*/OVA* without degradation was removed by centrifugation (10000 g, 10 min). The concentration of Zn^{2+} and Al^{3+} in the supernatant was detected by ICP-MS (PerkinElmer NexION 300X, USA).

Distribution and Accumulation of ZAlum/OVA in Vivo. Erythrocytes were heated for 20 min at 48 °C under continuous shaking, generating senescent RBCs (sRBCs).[7] To investigate the spleen accumulation of ZAlum/OVA, B16F10-OVA tumor-bearing mice were given different dosages (0, 1, 2, and 3 D; 1 D = 2×10^8 cells) of sRBCs 16 h in advance, then Cy5-labelled ZAlum/OVA was injected intravenously (i.v.). At 24 h post-injection, mice were sacrificed and major organs (liver and spleen) were isolated for ex vivo imaging using an In Vivo Imaging System FX Pro (VISQUE In Vivo Elite, Korea). To investigate the tumor accumulation of ZAlum/OVA, mice were given 3 D

of sRBCs 16 h in advance[7], then the Cy5-labelled ZAlum/OVA was injected. Mice were imaged by an In Vivo Imaging System at different times (8, 16, 20, and 24 h). At 24 h post-injection, mice were sacrificed and major organs (liver, heart, lung, kidneys, spleen) and tumors were isolated for ex vivo imaging. Fluorescence signals were quantitatively analyzed using VISQUE software (VISQUE In Vivo Elite, Korea). Besides, the accumulation of ZAlum/OVA in the spleen and tumor was further detected by ICP-MS (PerkinElmer NexION 300X, USA).

Cell Cytotoxicity and Apoptosis: B16F10-OVA and DC2.4 cells were seeded into 96-well plates (1×10^4 cells/well) overnight and then treated with ZAlum or Alum for 24 h. The cell viability was detected by Cell Counting Kit-8 (CCK-8) assay (Beyotime Biotechnology, China). To detect the cell apoptosis, B16F10-OVA cells were collected after incubation with OVA, Zn^{2+}+OVA, Alum/OVA, Zn^{2+}+Alum/OVA and ZAlum/OVA for 24 h, then stained by an Annexin V-FITC/PI kit (Beyotime, China) and analyzed by flow cytometry (Beckman DxFLEX, USA).

Cellular Uptake: DC2.4 and B16F10 cells (2×10^5 cells) were seeded into glass-bottom dishes overnight and then treated with Lumogallion-ZAlum or Lumogallion-Alum at a concentration of 500 μg mL^{-1} for 4 h. Cells were sequentially stained with TRITC-phalloidin and DAPI. In another parallel experiment, after incubation of 0.5, 4 and 24 h, cells were sequentially stained with LysoTracker Red and DAPI. Similarly, DC2.4 cells were seeded into glass-bottom dishes overnight and then treated with Cy5-ZAlum/OVA or Cy5-Alum/OVA to detect the cellular uptake of OVA. Images were taken immediately using a confocal laser scanning microscope (Zeiss LSM790, Germany).

Western-Blot Assays: For LC3B and P62 expressions, DC2.4 cells were seeded into 24-well plates at a density of 3×10^5 cells per well and treated with OVA, Zn^{2+}+OVA, Alum/OVA, Zn^{2+}+ Alum/OVA and ZAlum/OVA for 24 h, respectively. Cell lysates were loaded on SDS-PAGE gels and transferred to PVDF membranes (Millipore, USA). Indicated primary antibodies were incubated at 4°C overnight and an HRP-linked goat anti-rabbit IgG (Affinity, USA) was used as the secondary antibody. The blot signals were visualized by ECL using the ChemiDoc Touch Imaging System (Bio-Rad, USA).

Bio-TEM Imaging: DC2.4 cells were treated with Alum, ZAlum and Rapamycin (an autophagy inducer as positive control) for 24 h, then collected, washed and centrifuged to obtain cell pellets. Finally, ultrathin sections of DC2.4 cell pellets were observed by TEM (Hitachi HT7700, Japan).

Analysis of DC Maturation and Antigen Presentation in Vitro: DC2.4 cells (1×10^5 per well) were seeded into 24-well plates overnight and then treated with OVA, Zn^{2+}+OVA, Alum/OVA, Zn^{2+}+ Alum/OVA or ZAlum/OVA for 24 h. Cells were collected and then stained with PE-CD80 (BioLegend 104707), FITC-CD40 (BioLegend 102905) and APC-CD86 (BioLegend 105011) for 20 min at 4°C before being analyzed by flow cytometry (Beckman DxFLEX, USA). In another parallel experiment, after incubation of 24 h, cells were collected and stained with APC-anti-H-2K^b/SIINFEKL (BioLegend 141605) and PE-I-A/I-E (BioLegend 116625) before analysis.

ROS and JC-1 Detection: B16F10-OVA cells (1×10^5 per well) were seeded in glass-bottom dishes, then treated with PBS, OVA, Zn^{2+}+OVA, Alum/OVA, Zn^{2+}+ Alum/OVA or ZAlum/OVA for 24 h.

The level of ROS and mitochondrial membrane potential in cells were detected using ROS assay kit (S0033M, Beyotime, China) and mitochondrial membrane potential assay kit with JC-1 (C2003S, Beyotime, China), respectively. Similarly, E.G7-OVA cells (1×10^5 per well) were treated with PBS, OVA, Zn^{2+}+OVA, Alum/OVA, Zn^{2+}+ Alum/OVA or ZAlum/OVA, and the level of ROS was also detected using ROS assay kit (S0033M, Beyotime, China).

ATP Detection and Immunofluorescence Staining. B16F10-OVA cells (1×10^5 per well) and E.G7-OVA cells (1×10^5 per well) were seeded in a 24-well plate overnight, then treated with PBS, OVA, Zn^{2+}+OVA, Alum/OVA, Zn^{2+}+ Alum/OVA or ZAlum/OVA for 24 h. Cell lysates and cultures were collected for intracellular level of ATP detection using an ATP assay kit (S0026, Beyotime, China). Intracellular HMGB1 distribution and CRT exposure were tested using immunofluorescence analysis. B16F10-OVA cells were seeded in glass-bottom dishes, and then treated with PBS, OVA, Zn^{2+}+OVA, Alum/OVA, Zn^{2+}+ Alum/OVA or ZAlum/OVA for 24 h. The cells were washed with PBS, fixed with 4% paraformaldehyde and permeabilized with 0.1% Triton X-100 for 10 min. After incubation with 10% FBS for 1 h, the cells were incubated with anti-HMGB1 or anti-CRT antibody for 1 h, and then incubated with Cy3-conjugated secondary antibody and DAPI for another 45 min. Finally, the cells were observed using a CLSM (Zeiss LSM790, Germany).

In Vitro Phagocytosis Assay: For in vitro phagocytosis assays, DC2.4 cells were labelled with Cell Tracker Deep Red (0.5 mM; MX4110, MaoKang, China), and B16F10-OVA and E.G7-OVA tumor cells were labelled with Cell Tracker Green (0.5 mM; MX4107, MaoKang, China). B16F10-OVA or E.G7-OVA tumor cells (1×10^5) were incubated with PBS, OVA, Zn^{2+}+OVA, Alum/OVA, Zn^{2+}+

Alum/OVA or ZAlum/OVA for 24 h, and then were co-cultured with DC2.4 cells (1×10^5) for 4 h. After incubation, the percentage of phagocytosis was detected by flow cytometry (Beckman Cytoflex, USA).

Antitumor Efficacy in Vivo: Once the tumor volume reached ~50 mm^3, B16F10-OVA melanoma mice were divided into 7 groups, and subsequently treated as follows: control, Zn^{2+}+OVA (230 μg Zn^{2+}+ 100 μg OVA), Alum/OVA (i.t.) (122 μg Alum with 100 μg OVA), Zn^{2+}+Alum/OVA (i.t.) (230 μg Zn^{2+}+ 122 μg Alum with 100 μg OVA), ZAlum/OVA (i.t.) (1 mg ZAlum with 100 μg OVA), ZAlum/OVA (i.v.) (1 mg ZAlum with 100 μg OVA), sRBC+ZAlum/OVA (i.v.) (1 mg ZAlum with 100 μg OVA), Alum/OVA has the same aluminum content as ZAlum/OVA. For the group of sRBC+ZAlum/OVA (i.v.), sRBCs were injected 16 h in advance, and ZAlum/OVA was injected through i.v. on days 10 and 16. Other groups were also treated on days 10 and 16. The tumor volumes were recorded at a time interval of 2 days and the mice were sacrificed at day 20. At day 20, major organs were harvested and subjected to H&E staining and tumors were harvested and subjected to TUNEL staining. Besides, the CRT exposure, HMGB1 location of tumor sections and LC3 expression of the spleen sections were analyzed by immunohistochemistry. In another parallel experiment, E.G7-OVA tumor-bearing mice were divided into 4 groups, and subsequently treated as follows: control, Zn^{2+}+OVA, ZAlum/OVA (i.v.) and sRBC+ZAlum/OVA (i.v.). The mice were treated on days 10 and 16, and recorded the tumor volumes until day 20.

Analysis of Immune Cells: B16F10-OVA melanoma mice were vaccinated twice and then sacrificed on day 20. B16F10-OVA tumors and tdLNs collected from mice were digested in RPMI 1640 media containing 5% FBS, collagenase (0.5 mg mL^{-1}) and DNase I (0.1 mg mL^{-1}) for 1 h at 37°C.

The spleens were also polished into single-cell suspension. Then, cells were stained with fluorescence-labelled antibodies PE-CD3 (BioLegend 100205), FITC-CD4 (BioLegend 100406) and APC-CD8 (BioLegend 100712) to analyze the level of T cells in tumors. Similarly, the cells in tdLNs were stained by PE-CD11c (BioLegend 117307), APC-CD86 (BioLegend 105011), FITC-CD80 (BioLegend 104605), PE/Cy7-MHC-I (BioLegend 116519), PE-CD3 (BioLegend 100205) and APC-CD8 (BioLegend 100712) to analyze the maturation of DCs and activation of T cells. Splenocytes were also stained with PE-CD11c (BioLegend 117307), APC-CD86 (BioLegend 105011), FITC-CD80 (BioLegend 104605), PE/Cy7-anti-H-2K^{b}/SIINFEKL (BioLegend 141608), PE-CD3 (BioLegend 100205), APC-CD8 (BioLegend 100712), FITC-CD44 (BioLegend 103005) and PerCP-CD62L (BioLegend 104429) to analyze the level of DCs and T cells. Furthermore, splenocytes co-incubated with OVA or individualized tumor-associated antigens (iTAAs) of B16F10 tumor cells (100 μg mL^{-1}) for 48 h, then the cells were stained by PE-CD3 (BioLegend 100205), FITC-CD4 (BioLegend 100406), APC-CD8 (BioLegend 100712) and Pacific Blue-IFN-γ (BioLegend 505817) to analyze the level of antigen-specific T cells by flow cytometry (Beckman Cytoflex, USA).

Analysis of Cytokine Secretion in the Tumor Tissues. To examine IL-12 and TNF-α secretion in tumors, B16F10-OVA melanoma mice were vaccinated twice and then sacrificed on day 20. Then the tumors were collected and homogenized. IL-12 and TNF-α in the supernatants were measured using an ELISA kit.

Statistical Analysis: Data presented as the mean ± SD based on at least triplicate experiments were analyzed by one-way ANOVA with post multiple comparisons using Tukey’s test within GraphPad

Prism 9 software.

Supplementary References

[1] X. Yan, Z. Chen, Y. Wang, J. Zhang, Z. Zhou, H. Lv, *J. Power Sources* **2020**, *448*, 227412.

[2] M. Jannesari, O. Akhavan, H. R. Madaah Hosseini, B. Bakhshi, *J. Colloid Interface Sci.* **2023**, *637*, 237.

[3] Y. Sun, H. Guo, W. Zhang, T. Zhou, Y. Qiu, K. Xu, B. Zhang, H. Yang, *Ceram. Int.* **2016**, *42*, 9648.

[4] L. Zhang, Y. Jia, Y. Huang, H. Liu, X. Sun, T. Cai, R. Liu, Z. P. Xu, *Nano Res.* **2021**, *14*, 1326.

[5] I. Mile, A. Svensson, A. Darabi, M. Mold, P. Siesjö, H. Eriksson, *J. Immunol. Methods* **2015**, *422*, 87.

[6] Z. Liang, X. Wang, G. Yu, M. Li, S. Shi, H. Bao, C. Chen, D. Fu, W. Ma, C. Xue, B. Sun, *Nano Today* **2022**, *43*, 101445.

[7] I. Theurl, I. Hilgendorf, M. Nairz, P. Tymoszuk, D. Haschka, M. Asshoff, S. He, L. M. Gerhardt, T. A. Holderried, M. Seifert, S. Sopper, A. M. Fenn, A. Anzai, S. Rattik, C. McAlpine, M. Theurl, P. Wieghofer, Y. Iwamoto, G. F. Weber, N. K. Harder, B. G. Chousterman, T. L. Arvedson, M. McKee, F. Wang, O. M. Lutz, E. Rezoagli, J. L. Babitt, L. Berra, M. Prinz, M. Nahrendorf, G. Weiss, R. Weissleder, H. Y. Lin, F. K. Swirski, *Nat. Med.* **2016**, *22*, 945.
